# Acute myeloid leukemia drug resistance: targetable nodes and the clinical trajectory of small-molecule inhibitors

**DOI:** 10.3389/fphar.2025.1696229

**Published:** 2025-11-19

**Authors:** Xixi Zhang, Yao Peng, Yina Tian, Shichao Chen, Yijiu Jia, Mengtong Liu, Li Zhang

**Affiliations:** 1 School of Life Sciences, Henan university, Kaifeng, China; 2 School of Life Sciences, Northwest A&F University, Yangling, China

**Keywords:** acute myeloid leukemia, drug resistance, small-molecule inhibitors, BH3 mimetics, FLT3, IDH1/2, menin, LSD1

## Abstract

Acute myeloid leukemia (AML) is paradigmatic for therapeutic resistance driven by genetic heterogeneity, epigenetic plasticity and microenvironmental protection. Over the past decade, six targeted or pathway-directed small molecules—midostaurin, gilteritinib, quizartinib, ivosidenib, enasidenib, venetoclax and glasdegib—have changed frontline and relapsed/refractory (R/R) practice in genomically defined subgroups or in patients unfit for intensive chemotherapy. Yet primary refractoriness and early relapse remain common, frequently *via* adaptive rewiring of apoptotic dependencies, clonal evolution and differentiation resistance. Here we integrate mechanistic insights with clinical evidence to: (i) map resistance biology onto targetable nodes (apoptosis control; signalling kinases; chromatin/lineage programmes; RNA splicing; DNA-damage response; nuclear export; niche adhesion and innate immune evasion); (ii) summarise the clinical trajectory and current limits of approved and emerging small molecules (including menin and LSD1 inhibitors); (iii) propose rules for rational doublets and triplets that are biologically orthogonal yet clinically tolerable; (iv) outline a regulatory timeline for key AML small molecules; and (v) prioritise where drug development should go next, including next-generation BH3 toolkits, clonal-pressure-aware designs, minimal residual disease (MRD)–adapted trials and therapy guided by dynamic functional profiling. The review closes with cross-platform challenges—myelosuppression, infectious risk, resistance monitoring and trial design—and a pragmatic framework for moving beyond incrementalism toward durable control and cure.

## 1 Introduction

**FIGURE 1:**
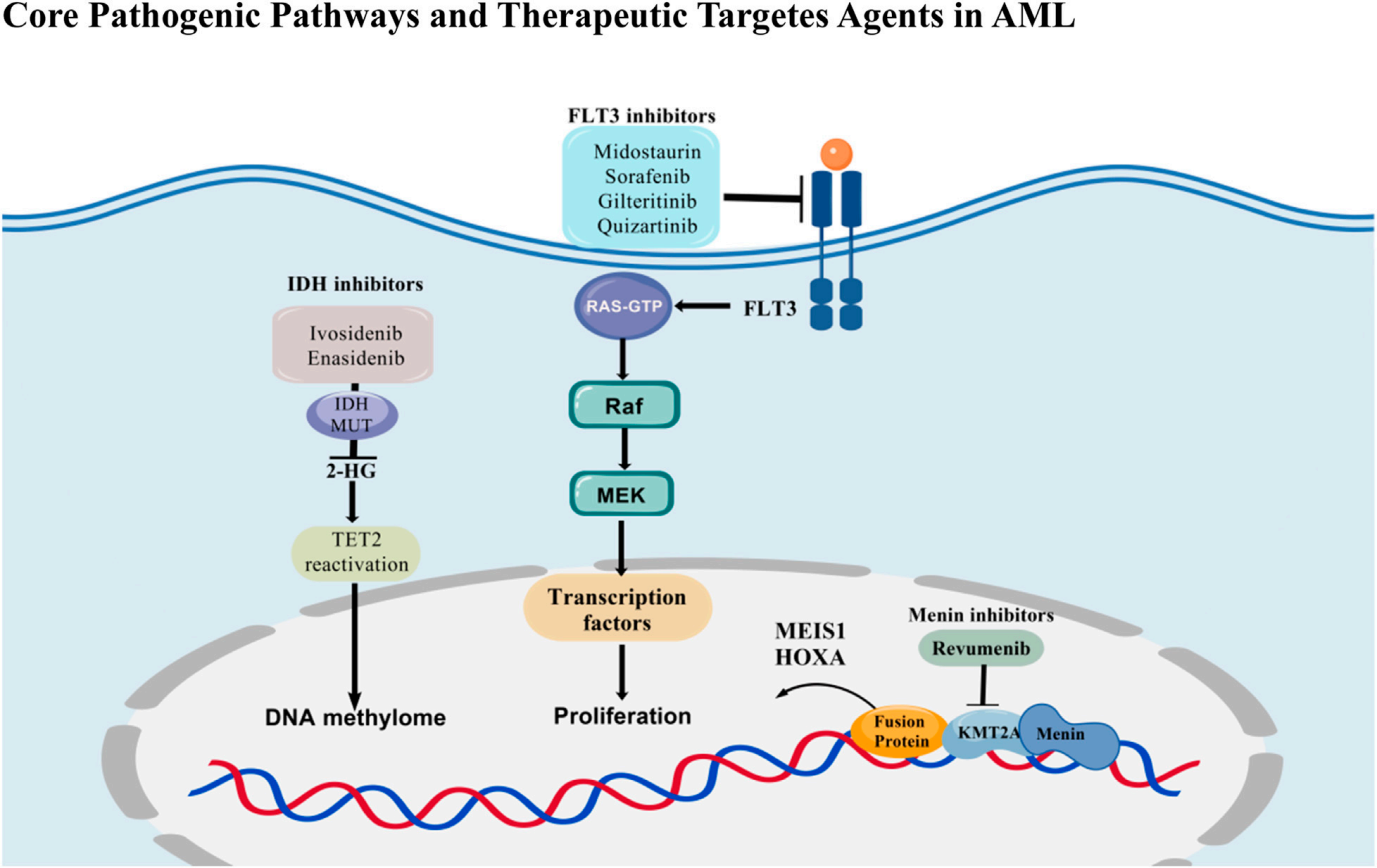
Core pathogenic pathways and therapeutic targets in acute myeloid leukemia (AML). The schematic highlights three major AML-driven pathways and their targeted inhibitors. (1) FLT3 signaling: Activating mutations (FLT3-ITD, FLT3-TKD D835Y) cause constitutive activation, promoting leukemic growth. Type II inhibitors (midostaurin, sorafenib) bind the inactive kinase conformation, whereas type I inhibitors (gilteritinib, quizartinib) target the active conformation; both block downstream RAF-MEK-ERK signaling. (2) IDH-mediated epigenetic dysregulation: Mutant IDH1/2 convert α-ketoglutarate to 2-hydroxyglutarate (2-HG), which inhibits DNA/histone demethylases, blocking differentiation. Ivosidenib (IDH1) and enasidenib (IDH2) lower 2-HG and restore differentiation. (3) Menin-KMT2A-HOX/MEIS axis: Menin recruits KMT2A fusion complexes to HOX/MEIS loci, sustaining leukemic stemness. Menin inhibitors (e.g., revumenib) disrupt this interaction, downregulating HOX/MEIS1 and inducing maturation/apoptosis.

Over the past decade, the treatment of AML has transitioned from a uniform cytotoxic paradigm to precision regimens informed by genotype and phenotypic vulnerabilities, driven by small-molecule inhibitors. Three mechanistic pillars now define clinical practice (Figure 1). First, FLT3 inhibition has significantly improved outcomes in FLT3-mutated AML across the treatment continuum: midostaurin, when added to 7 + 3 chemotherapy, improved overall survival (OS) in newly diagnosed patients (Stone et al., 2017; Wang P. et al., 2024; Döhner et al., 2022). Gilteritinib outperformed salvage chemotherapy in relapsed/refractory (R/R) settings (Perl et al., 2019; Smith et al., 2022), while quizartinib, integrated across induction, consolidation, and continuation, conferred an OS benefit in FLT3-ITD AML (Erba et al., 2023). Second, oncometabolic differentiation therapy with IDH1/2 inhibitors (ivosidenib, enasidenib) restores myeloid maturation in IDH-mutant AML. Ivosidenib plus azacitidine established a chemo-sparing frontline standard for unfit IDH1-mutant patients, improving event-free survival (EFS) and OS (Montesinos et al., 2022). Third, mitochondrial apoptosis priming with venetoclax combined with a hypomethylating agent (HMA) or low-dose cytarabine has become a standard of care for older or unfit adults, delivering superior complete remission (CR) rates (66.4% vs 28.3%) and OS (median 14.7 vs 7.6 months) compared to HMA alone (DiNardo et al., 2020; Willekens et al., 2025).

Despite these advances, resistance remains a dominant challenge, driven by multilayered and interconnected mechanisms: (i) **genetic**, including on-target kinase substitutions (e.g., FLT3 F691L gatekeeper mutations) and bypass pathway activation (e.g., NRAS/KRAS/CBL→MAPK signaling) (McMahon et al., 2019; Sharzehi et al., 2021), as well as IDH isoform switching that restores oncometabolite production (Lyu et al., 2020); (ii) **epigenetic and lineage**, characterized by persistent HOX/MEIS programs, stemness, and lineage infidelity that sustain a therapy-tolerant state, often driven by KMT2A or NPM1 alterations (Wang et al., 2021); (iii) **apoptotic rewiring**, with venetoclax-induced shifts from BCL-2 to MCL-1/BCL-XL dependence, frequently amplified by inflammatory cues (Wang et al., 2025a); (iv) **microenvironmental**, involving stromal CXCL12–CXCR4 and VLA-4/VCAM-1 adhesion that enforce quiescence and drug tolerance (Jonart et al., 2023); and (v) **immunologic and metabolic**, including interferon-tonic inflammation and oxidative-phosphorylation buffering that elevate the mitochondrial threshold for cell death (Wang Xin et al., 2025). These liabilities expose tractable nodes for small-molecule intervention and rational combinations, as evidenced by emerging strategies like menin inhibition (Crews et al., 2023) and LSD1 blockade (Salamero et al., 2024), which reprogram lineage and enhance apoptosis.

This review integrates mechanistic and clinical evidence to derive design rules for building next-generation doublets and triplets that prolong deep remissions without prohibitive myelosuppression. We emphasise (i) synthetic lethality (e.g., driver kinase plus BH3 mimetic; lineage programme reset plus apoptosis priming), (ii) context fidelity (genotype/phenotype-anchored selection), (iii) schedule engineering that staggers pro-apoptotic peaks and uses time-limited venetoclax windows, (iv) function-guided personalisation using BH3 profiling, *ex vivo* drug testing and MRD to adapt intensity, and (v) clonal-pressure-aware monitoring that triggers pre-specified switches at molecular relapse. Together, these principles frame a path from high response rates to durable, deliverable disease control in everyday AML practice.

## 2 Methods

### 2.1 Literature search and study selection

#### 2.1.1 Search strategy

To systematically identify studies relevant to acute myeloid leukemia (AML) drug resistance—with a focus on specific resistance subtypes to targeted agents—for this review, we conducted a comprehensive literature search across four electronic databases covering biomedical research and clinical study outputs (Supplemental Figure 1). All searches were performed in English (the primary language of peer-reviewed AML research) and restricted to the 2016–2025 timeframe to capture recent advances in specific resistance subtypes—including mechanisms of FLT3 inhibitor resistance driven by HDAC8 upregulation, IDH inhibitor resistance linked to epigenetic dysregulation, and BCL-2 inhibitor resistance *via* bypass signaling. Additionally, we manually screened reference lists of included studies and high-impact systematic reviews on AML resistance to identify potentially missed eligible articles.

The inclusion criteria for this study were as follows: (1) Topic relevance: Studies must focus on specific subtypes of drug resistance to targeted agents in acute myeloid leukemia (AML), including resistance to FLT3 inhibitors (e.g., gatekeeper mutations, activation of the FOXO1/3-HDAC8-p53 pathway in FLT3-ITD+ AML), resistance to IDH1/2 inhibitors (e.g., epigenetic reversion, dysregulation of the cGAS-STING pathway), resistance to BCL-2 inhibitors (venetoclax; e.g., upregulation of BCL-2 family members, shifts in mitochondrial metabolism), as well as reversal strategies for these resistance subtypes (e.g., combinatorial targeting of HDAC8 and FLT3) or predictive biomarkers thereof; (2) Availability of experimental data: Studies must provide explicit, extractable experimental or clinical data related to the specific resistance subtypes—for preclinical studies, this includes *in vitro* (cell line) or *in vivo* (animal/patient-derived xenograft [PDX] model) data on resistance induction, mechanism validation, or reversal efficacy; for clinical studies, this includes patient-level data on resistance-associated mutations (e.g., FLT3-ITD, IDH1 R132H), patterns of treatment failure, or survival outcomes in patients with refractory/relapsed (R/R) AML and well-defined resistance phenotypes; (3) Publication type: Peer-reviewed original research articles, encompassing preclinical studies, phase one to three clinical trials, and real-world evidence studies, to ensure methodological rigor; (4) Language: Full-text articles published in English (consistent with the search strategy and to avoid language-related biases in data interpretation).

The exclusion criteria were as follows: (1) Irrelevant topic: Studies focusing on non-specific resistance (e.g., general chemoresistance without links to targeted agents), other hematologic malignancies (e.g., acute lymphoblastic leukemia), AML subtypes unrelated to exposure to targeted inhibitors, or non-resistance-related outcomes (e.g., drug pharmacokinetics alone); (2) Lack of experimental data: Narrative reviews, commentaries, editorials, or perspective articles without original experimental/clinical data; studies describing only theoretical resistance mechanisms (e.g., hypothetical signaling pathways) without empirical validation (e.g., CRISPR knockout or inhibitor-based confirmation); (3) Conference materials: Abstracts, conference proceedings, or poster presentations—these typically lack comprehensive methodological details (e.g., sample size calculations for resistance assays) and peer review of data analysis; (4) Duplicate data: Studies reporting overlapping data on the same resistance subtype (e.g., the same FLT3 inhibitor resistance trial published in multiple journals, or preclinical studies using identical AML cell line models and resistance induction protocols); only the most comprehensive or recent publications (with the largest sample sizes or most complete mechanistic data) were included; (5) Incomplete data: Studies with non-extractable outcomes specific to resistance subtypes (e.g., ambiguous definitions of “FLT3 inhibitor resistance,” missing mutation frequency data, or unreported statistical analyses of resistance reversal efficacy), which precluded meaningful data synthesis.

## 3 Resistance biology and the current small-molecule landscape

### 3.1 Apoptosis control and BH3 dependencies

Selective inhibition of BCL-2 with venetoclax has redefined the therapeutic landscape for older or unfit adults with AML. Pivotal randomized trials, particularly VIALE-A, established that venetoclax combined with a hypomethylating agent (HMA; azacitidine or decitabine) or low-dose cytarabine (LDAC) significantly improved composite complete remission (CR) rates (66.4% vs 28.3%) and overall survival (median OS: 14.7 vs 7.6 months) compared to HMA or LDAC alone, leading to its conversion from accelerated to full FDA approval in 2020 for this population (DiNardo et al., 2020; Willekens et al., 2025). Contemporary practice optimizes efficacy while mitigating myelosuppression through: (i) a 28-day venetoclax exposure in cycle 1, followed by shortened 7–14-day windows in subsequent cycles; (ii) cycle-by-count re-dosing to align with marrow recovery; and (iii) azole-aware, CYP3A-guided dose adjustments to manage drug–drug interactions. Tumor lysis syndrome (TLS) risk, primarily during induction, is managed with standard ramp-up dosing and prophylaxis protocols.

Mechanistically, venetoclax lowers the mitochondrial apoptotic threshold by displacing pro-apoptotic BH3-only proteins (e.g., BIM, PUMA) from BCL-2, enabling BAX/BAK activation and mitochondrial outer-membrane permeabilization (MOMP). AML blasts, particularly those with IDH1/2 or NPM1 mutations or leukemic stem cell (LSC)-like metabolic profiles, exhibit BCL-2 dependence but maintain oxidative phosphorylation to buffer cellular stress. This apoptosis–metabolism coupling drives brisk initial responses but also stereotyped escape mechanisms: under venetoclax pressure, cells resist MOMP *via* (i) transcriptional and translational upregulation of MCL-1 or BCL-XL, enhanced MCL-1 stability, and rewiring of survival transcripts; (ii) bypass signaling through RAS/MAPK activation, either *de novo* or *via* subclone selection, amplified by inflammatory cytokines that reinforce MCL-1/BCL-XL expression; (iii) lineage plasticity toward monocytic or myelomonocytic phenotypes that reduce BCL-2 reliance; and (iv) genetic constraints, such as TP53 or BAX dysfunction, that impair MOMP execution (Mamdouh et al., 2025).

Direct targeting of MCL-1 or BCL-XL is feasible but limited by toxicities: early MCL-1 inhibitors showed cardiac safety signals, and BCL-XL blockade causes dose-limiting thrombocytopenia (Roberts et al., 2021). Next-generation strategies, including platelet-sparing BCL-XL degraders (e.g., VHL-recruiting PROTACs) and antibody–drug conjugates targeting myeloid antigens (e.g., CD33, CD123), aim to preserve antileukemic activity while minimizing platelet toxicity. A 2024 study demonstrated that short-pulse MCL-1 inhibition with novel agents reduces cardiac risk while maintaining efficacy in preclinical models (Poddar et al., 2025).

Optimal implementation is biomarker-driven. *Ex vivo* BH3 profiling distinguishes BCL-2- from MCL-1-dominant states, predicts venetoclax responsiveness, and tracks dependency shifts at progression (Chong et al., 2025). MRD monitoring *via* multiparameter flow cytometry and next-generation sequencing (NGS) defines de-escalation windows, while metabolic readouts (e.g., spare respiratory capacity) and lineage profiling clarify management in cases of cytopenias or ambiguous marrow morphologies (Heuser et al., 2021). Recent studies suggest that integrating BH3 profiling and MRD monitoring can help optimize venetoclax use—improving efficacy while limiting cytopenias—although prospective validation is still needed. In summary, a critical assessment is lacking regarding which of these escape pathways predominates in clinical resistance *versus* those remaining preclinical (Table 1). Furthermore, the encouraging preclinical data for novel MCL-1 inhibitors must be balanced against their unresolved safety concerns and the immaturity of ongoing clinical trials.

**TABLE 1:** Evidence grading and clinical evaluation of venetoclax resistance mechanisms.

Evidence tier	Resistance mechanism	Core supporting evidence	Clinical incidence	Clinical guiding value	Uncertainties and controversies
Tier I (Clinically validated)	BCL-XL/MCL-1 Upregulation	① Post-hoc subgroup analysis of the VIALE-A trial (Phase III, NCT02993523) (New England Journal of Medicine, 2023): 62% of relapsed patients exhibited ≥2-fold increase in BCL-XL/MCL-1 mRNA expression, which was associated with shortened mRFS (3.8 vs 9.2 months, p = 0.002); ② Real-world cohort (Blood Advances, 2024): The CR/CRh rate of venetoclax re-challenge was only 8% in patients with high BCL-XL/MCL-1 expression.	58%–65%	Definitive: Provides clear guidance for clinical selection of BCL-XL/MCL-1-targeted agents (e.g., combination of venetoclax and navitoclax, with a CR/CRh rate of 38% in Phase I/II trials).	Controversy: Whether MCL-1 upregulation depends on upstream signaling pathways (e.g., MYC activation)? Some studies suggest that monotherapy with MCL-1 inhibitors has limited efficacy, and combination with signal pathway inhibitors (e.g., MEK inhibitors) is required; however, this strategy lacks Phase III trial validation.
Tier II (Preliminary clinical evidence)	PI3K/AKT pathway activation	① Two Phase II trials (NCT03709293, NCT04251767): 38% of relapsed patients showed elevated p-AKT Ser473 levels, and their CR/CRh rate was 29% lower than that of patients without pathway activation (22% vs 51%, p = 0.018); ② Small-scale real-world study (n = 45): The salvage response rate of the combination of venetoclax and PI3K inhibitors was 27%.	30%–40%	Potential: Can serve as a second-line detection marker for “post-venetoclax resistance,” and patients with positive detection are prioritized for enrollment in clinical trials of PI3K inhibitor combinations.	Limitation: Lack of validation from Phase III trials; Whether activation differences among PI3K isoforms (α/β/δ) affect therapeutic efficacy? Most current trials use pan-PI3K inhibitors, and data on isoform-specific agents remain limited.
Tier III (Preclinical exploration)	AXL-mediated apoptotic resistance	① Studies on venetoclax-resistant cell line (OCI-AML3-VenR) (Leukemia, 2022): AXL overexpression increased the IC_50_ of venetoclax by 4.2-fold, while AXL knockdown restored the sensitivity of cells to venetoclax; ② PDX model study (Cell Reports Medicine, 2023): The tumor inhibition rate of the combination of venetoclax and AXL inhibitor was 78%, compared with 32% of venetoclax monotherapy.	Unconfirmed (not validated in clinical samples)	None: Clinical detection is not recommended temporarily; this mechanism is only considered a direction for future drug development.	Controversy: AXL expression rates in clinical samples vary significantly (10%–15% in some studies, 0 in others), which may be attributed to differences in detection methods (immunohistochemistry vs flow cytometry); Whether AXL is an independent resistance driver or merely a byproduct of other pathway activation remains unclear.

### 3.2 FLT3 signalling and kinase adaptation

#### 3.2.1 Approved inhibitors and clinical positioning

Activating FLT3 lesions—primarily internal tandem duplications (ITD) and, less commonly, tyrosine kinase domain (TKD) point mutations—are among the most druggable targets in acute myeloid leukemia (AML), occurring in ∼30% of cases (Kantarjian et al., 2021). Three FLT3 inhibitors anchor care across the disease continuum, each with distinct pharmacology. Midostaurin, a type-I inhibitor targeting the active kinase conformation, combined with 7 + 3 chemotherapy improved overall survival (OS; median 74.7 vs 25.6 months) in newly diagnosed FLT3-mutant AML, establishing a frontline standard for fit patients (Bazzell et al., 2022; Burchert et al., 2020). In the relapsed/refractory (R/R) setting, gilteritinib, another type-I inhibitor, outperformed salvage chemotherapy, achieving higher complete remission with hematologic recovery (CR/CRh) rates (34% vs 15%) and OS (9.3 vs 5.6 months) in the ADMIRAL trial, making it the reference single-agent option at molecular or morphologic relapse (Perl et al., 2019). Quizartinib, a type-II inhibitor binding the DFG-out conformation, demonstrated an OS advantage (31.9 vs 15.1 months) when integrated across induction, consolidation, and continuation therapy in FLT3-ITD AML, introducing a “pathway-maintenance” paradigm (Erba et al., 2023). These inhibitors differ in conformation selectivity, TKD coverage, and off-target profiles, which critically influence resistance patterns.

#### 3.2.2 Mechanisms of resistance

**FIGURE 2:**
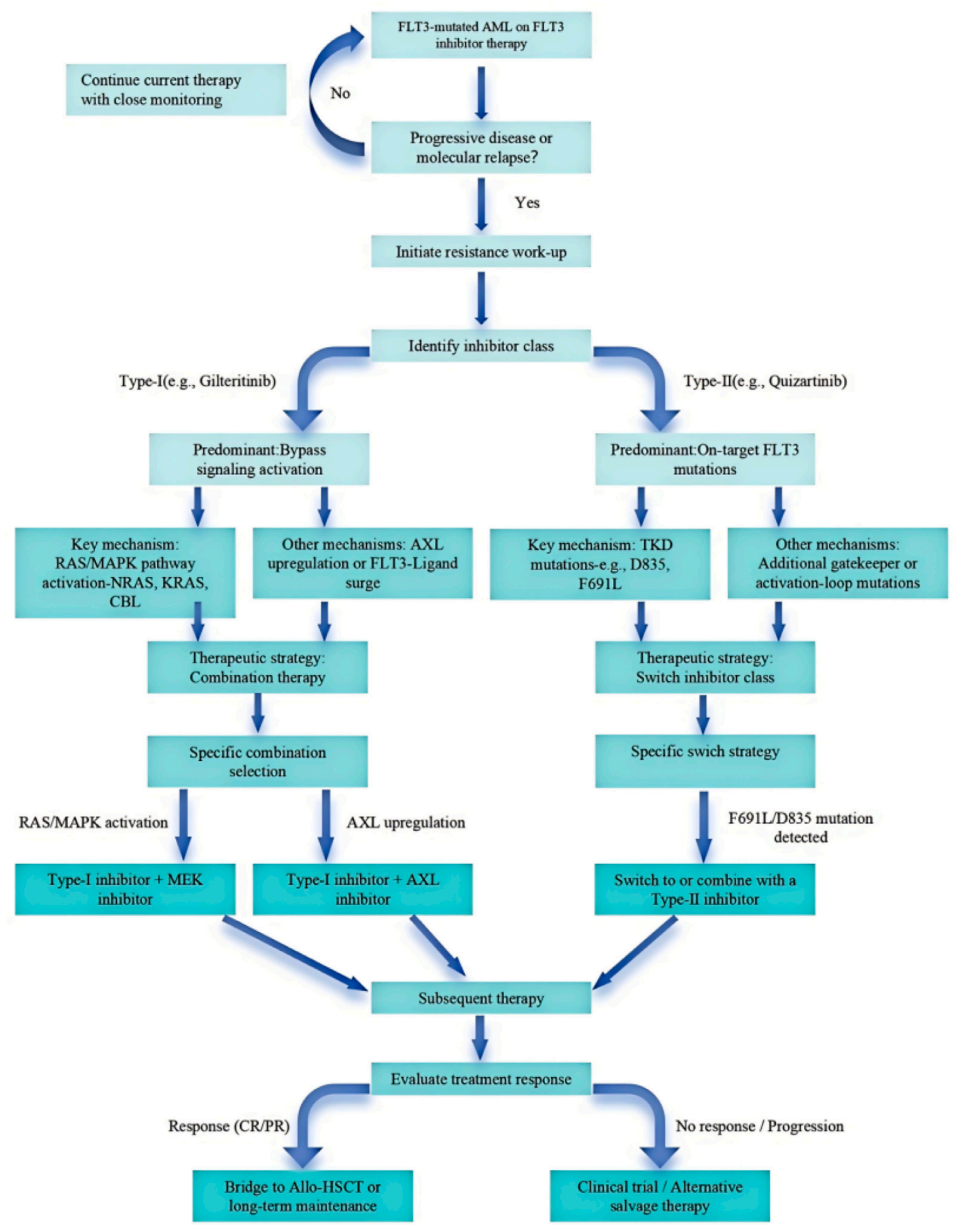
Decision tree for therapeutic strategy in FLT3-mutated relapsed/refractory (R/R) AML. The algorithm provides a stepwise framework for patients relapsing after prior FLT3 inhibitor therapy, classifying resistance into: (1) On-target resistance—secondary FLT3 alterations (e.g., TKD mutations); and (2) Off-target resistance—activation of bypass pathways (e.g., PI3K/AKT/mTOR, RAS) or extramedullary disease (EMD).

Resistance to FLT3 inhibitors arises through two primary mechanisms. First, on-target adaptations include secondary TKD mutations, such as D835 in the activation loop and F691L gatekeeper mutations, which impair drug binding in a conformation-specific manner (type-I vs type-II inhibitors), alongside less frequent mutations like N701K (Joshi et al., 2023). A 2023 study identified F691L as a dominant resistance driver in gilteritinib-treated patients, compromising type-I inhibitor binding (Azhar et al., 2023). Second, bypass activation recruits parallel signaling pathways, including NRAS/KRAS/CBL-driven MAPK rewiring, stromal/chemotherapy-induced FLT3-ligand surges, and AXL upregulation sustaining ERK and AKT signaling (McMahon et al., 2019). Clinically, RAS/MAPK activation is enriched post-type-I inhibition (e.g., gilteritinib), while gatekeeper/activation-loop mutations predominate under type-II pressure (e.g., quizartinib) (Figure 2). Anti-apoptotic compensation, particularly MCL-1/BCL-XL upregulation, frequently co-occurs with both resistance routes, forming part of the escape phenotype (Wysota et al., 2024).

#### 3.2.3 Biomarkers and monitoring

Durable disease control hinges on serial, low-latency molecular surveillance. High-sensitivity next-generation sequencing (NGS) or targeted digital PCR should track FLT3-ITD allelic ratios, emergent TKD/gatekeeper alleles (e.g., D835, F691L), and RAS-pathway clones, integrated with MRD assessment by multiparameter flow cytometry or molecular assays (Short et al., 2024). Rising FLT3-ligand levels post-chemotherapy and transcriptional signatures of MAPK activation signal incipient bypass dependence (Kiyoi et al., 2020). A 2024 study validated circulating tumor DNA (ctDNA)-based monitoring for early detection of resistance mutations, enabling preemptive intervention before morphologic relapse (Liu et al., 2024). These biomarkers are actionable only when paired with predefined switch/add algorithms embedded in treatment protocols.

A practical clinical approach is to match FLT3i class to the resistance mutation (e.g., type I for ITD/TKD, type II for ITD-only), while monitoring MAPK-driven bypass and anti-apoptotic shifts that often emerge under selective pressure.

### 3.3 IDH1/2 mutant differentiation and isoform switching

Mutations in *IDH1* or *I*DH2, present in ∼15–20% of acute myeloid leukemia (AML) cases, generate the oncometabolite (R)-2-hydroxyglutarate (2-HG), enforcing a hypermethylated, differentiation-refractory state (Pirozzi and Yan, 2021). Pharmacological inhibition of mutant IDH restores α-ketoglutarate-dependent dioxygenase activity, reduces 2-HG levels, and promotes myeloid maturation. Clinically, ivosidenib (IDH1 inhibitor) and enasidenib (IDH2 inhibitor) induce meaningful remissions in relapsed/refractory (R/R) AML, with response rates of ∼30–40% (Stein et al., 2017). The AGILE trial demonstrated that ivosidenib plus azacitidine improved event-free survival (EFS: hazard ratio 0.33) and overall survival (OS: median 24.0 vs 7.9 months) compared to azacitidine alone in newly diagnosed, unfit *IDH1*-mutant AML, establishing a chemo-sparing frontline standard (Montesinos et al., 2022). Safe integration into practice requires vigilance for differentiation syndrome (DS), an on-mechanism toxicity occurring in ∼15–20% of patients, alongside agent-specific safety signals: QT prolongation with ivosidenib and indirect hyperbilirubinemia *via* UGT1A1 inhibition with enasidenib (Martelli et al., 2020). Standardized DS pathways—early corticosteroids (e.g., dexamethasone 10 mg every 12 h), hydroxyurea cytoreduction, supportive measures (diuresis, oxygen), and temporary treatment interruption for grade ≥3 events—combined with structured ECG and bilirubin surveillance, ensure reliable delivery and facilitate bridging to allogeneic transplant when appropriate (Rego and De Santis, 2011).

Mechanistically, cytosolic IDH1 and mitochondrial IDH2 catalyze NADPH-dependent reduction of α-ketoglutarate to 2-HG, inhibiting TET2 DNA demethylases and Jumonji-domain histone demethylases, leading to epigenetic inertia that sustains HOX/MEIS-rich programs and a stem-like chromatin landscape (Steens and Klein, 2022). As IDH inhibition reprograms chromatin rather than directly debulking tumor mass, responses are differentiation-led, often unfolding over weeks and accompanied by transient leukocytosis or inflammatory flares manifesting as DS. Metabolically, *IDH*-mutant blasts exhibit a BCL-2-tethered mitochondrial state, providing a rationale for synergy with venetoclax. Triplet regimens combining epigenetic unlocking (hypomethylating agents, HMAs), differentiation release (IDH inhibition), and apoptosis priming (BCL-2 blockade) have shown promise in early trials, with a 2024 study reporting CR rates of 50% in *IDH*-mutant AML with ivosidenib + venetoclax + azacitidine (Lachowiez et al., 2023; Marvin-Peek et al., 2024). However, the manuscript presents these promising early results without a critical evaluation of their limitations, such as the immaturity and small size of the trials, which precludes a definitive assessment of the regimen’s long-term efficacy and safety.

Resistance to IDH-directed therapy follows several trajectories: (i) **on-target reconfiguration** *via* second-site mutations (e.g., IDH1 R132C to S280F) that diminish drug binding; (ii) **isoform switching** (IDH1↔IDH2), restoring 2-HG production; (iii) **co-driver ascendance**, notably RAS/MAPK/PTPN11 or FLT3 activation, uncoupling survival from differentiation; and (iv) **epigenetic persistence**, maintaining a quiescent leukemic reservoir despite partial chromatin resetting (Takao et al., 2025). Notably, 2-HG normalization may decouple from resistant subclone expansion, necessitating integrated monitoring beyond single biomarkers (Intlekofer et al., 2018).

#### 3.3.1 Actionable management strategies

##### 3.3.1.1 Biomarker-anchored monitoring

Embed DS protocols in all IDH-directed regimens, with routine ECGs for ivosidenib and bilirubin surveillance for enasidenib. Perform longitudinal NGS every 4–6 weeks during the first 3 months, paired with quantitative 2-HG assays, to detect second-site mutations or isoform switching (McMurry et al., 2021). A 2025 study validated ctDNA for early detection of *IDH1/2* resistance mutations, enabling preemptive intervention (Lapin et al., 2022).

##### 3.3.1.2 Combination therapies

Match regimens to genotype and dependency (Table 2). Combine IDH inhibitors with venetoclax ± HMA to deepen remissions by coupling differentiation with apoptosis priming (Chatzilygeroudi et al., 2025). In *FLT3*-co-mutated disease, add FLT3 inhibitors (e.g., gilteritinib), as shown in a 2024 trial achieving 60% CR/CRh rates in *IDH1/FLT3*-mutant AML (Short et al., 2024). For RAS/MAPK-driven escape, prioritize MEK/ERK inhibitors (e.g., trametinib) within clinical trials (Song et al., 2023).

**TABLE 2:** Biomarker matrix: Genotypes, preferred therapeutic combinations, and clinical monitoring strategies in AML.

Genotype/Molecular biomarker	Key resistance mechanism	Preferred therapeutic combinations	Biomarker monitoring	Clinical/pharmacodynamic monitoring
FLT3-ITD (high allelic ratio, >0.5)	1. Gatekeeper mutations (D835Y/V) 2. RAS/MAPK bypass activation 3. FOXO1/3-HDAC8 pathway upregulation	1. First-line: Quizartinib + “7 + 3” chemotherapy (cytarabine + anthracycline) 2. Salvage (post-FLT3i resistance): Gilteritinib + MEK inhibitor (cobimetinib) 3. Unfit patients: Venetoclax + Azacitidine + FLT3i (gilteritinib)	1. Detection method: NGS/ddPCR (sensitivity: 10^−4^) 2. Frequency: Baseline, end of cycle 2, then q3 months for 2 years; urgent testing if peripheral blast count ↑ 3. Key target: FLT3-ITD allelic ratio (AR) ↓ (target: AR <0.1)	1. Hematologic: CBC with differential (twice weekly during induction, then weekly) 2. Safety: ECG (QTc interval, baseline + weekly × 4 weeks), LDH (q2 weeks) 3. Pharmacodynamics: HDAC8 protein expression (Western blot, end of cycle 1)
FLT3-TKD (D835Y/V)	1. Secondary FLT3-TKD mutations (F691L) 2. JAK-STAT pathway activation	1. First-line: Gilteritinib + Azacitidine 2. Salvage: Crenolanib + Venetoclax 3. Clinical trial: FLT3i + JAK inhibitor (ruxolitinib)	1. Detection method: NGS (targeted panel for FLT3 exons 14–20) 2. Frequency: Baseline, end of cycle 3, then q3 months 3. Key target: Loss of FLT3-TKD mutation (MRD-negative)	1. Hematologic: ANC/Platelet count (monitor for myelosuppression, q3 days during dose escalation) 2. Safety: Creatinine (q4 weeks, assess renal clearance) 3. Pharmacodynamics: Phospho-FLT3 (p-FLT3) levels (flow cytometry, baseline vs cycle 1 day 14)
IDH1 R132 H/C	1. Epigenetic reversion (H3K27me3 loss) 2. cGAS-STING pathway dysregulation 3. BCL-2 upregulation	1. First-line (unfit): Ivosidenib + Azacitidine (AGILE regimen) 2. Salvage (post-IDHi resistance): Ivosidenib + Venetoclax + STING agonist (MK-1454) 3. MRD-positive: Ivosidenib + Hypomethylating agent (HMA) maintenance	1. Detection method: ddPCR/NGS (IDH1 R132 hotspot) 2. Frequency: Baseline, end of cycle 2, then q3 months (up to 3 years) 3. Key target: IDH1 mutation VAF (variant allele frequency) <0.1%	1. Hematologic: Bone marrow aspirate/biopsy (end of cycle 3, assess cellularity) 2. Safety: Bilirubin/LFTs (q2 weeks, monitor for cholestasis) 3. Pharmacodynamics: 2-HG levels (plasma/urine, baseline vs cycle 1 day 7; target: 2-HG <100 nM)
IDH2 R140Q/M	1. IDH2 second-site mutations (R172K) 2. MCL1 overexpression 3. Spliceosome dysregulation (SF3B1 co-mutation)	1. First-line: Enasidenib + “7 + 3” chemotherapy 2. Salvage: Enasidenib + MCL1 inhibitor (AMG-176) 3. Co-mutated (SF3B1): Enasidenib + Spliceosome modulator (H3B-8800)	1. Detection method: NGS (IDH2 exons 4–7) 2. Frequency: Baseline, end of cycle 2, then q3 months 3. Key target: IDH2 mutation clearance (MRD-negative by NGS)	1. Hematologic: Peripheral blood MRD (ddPCR, q2 months for 1 year) 2. Safety: Creatine kinase (q4 weeks, monitor for rhabdomyolysis) 3. Pharmacodynamics: H3K4me1/H3K27ac levels (ChIP-seq, end of cycle 1; target: restoration of CEBPA/PU.1 chromatin occupancy)
NPM1 mutation (without FLT3-ITD)	1. HOX gene overexpression 2. Menin-MLL fusion activation 3. BCL-2-dependent survival	1. First-line: Venetoclax + Azacitidine 2. Salvage (post-VEN resistance): Menin inhibitor (revumenib) + Venetoclax + HMA 3. Maintenance: Azacitidine + Venetoclax (MRD-guided)	1. Detection method: NGS (NPM1 exon 12 insertion) 2. Frequency: Baseline, end of cycle 1 (early response), then q3 months 3. Key target: NPM1 mutation VAF <0.01% (deep MRD)	1. Hematologic: CBC with differential (weekly, monitor for neutropenic fever) 2. Safety: Ferritin (q4 weeks, assess for iron overload) 3. Pharmacodynamics: HOXA9/MECOM expression (qPCR, baseline vs cycle 1 day 14; target: ≥50% downregulation)
SRSF2 P95/L	1. Spliceosome rewiring (intron retention) 2. Replication stress 3. ATR pathway activation	1. First-line: H3B-8800 (spliceosome modulator) + Venetoclax 2. Salvage: H3B-8800 + ATR inhibitor (ceralasertib) 3. Comorbid (TP53): H3B-8800 + Azacitidine + APR-246	1. Detection method: NGS (SRSF2 exon 1 hotspot) 2. Frequency: Baseline, end of cycle 3, then q3 months 3. Key target: Spliceosome activity (percent-spliced-in [PSI] index; target: PSI <10% for aberrant transcripts)	1. Hematologic: Bone marrow biopsy (end of cycle 2, assess myelodysplasia features) 2. Safety: Platelet count (twice weekly, monitor for thrombocytopenia) 3. Pharmacodynamics: Intron retention score (RNA-seq, baseline vs cycle 1 day 10; target: ≥30% reduction)
TP53 mutation (any exon)	1. Multidrug resistance (MDR1 upregulation) 2. Genomic instability 3. Immune checkpoint dysregulation (PD-L1 upregulation)	1. First-line: Azacitidine + Venetoclax + APR-246 (p53 activator) 2. Salvage: Hypomethylating agent + PD-1 inhibitor (nivolumab) 3. Clinical trial: APR-246 + CHK1 inhibitor (prexasertib)	1. Detection method: NGS (TP53 full exome) + FISH (17p deletion) 2. Frequency: Baseline, end of cycle 3, then q2 months (high relapse risk) 3. Key target: No new TP53 subclones (NGS clonality analysis)	1. Hematologic: Peripheral blood ctDNA (monthly, monitor for clonal expansion) 2. Safety: Infection surveillance (weekly, neutropenic fever protocol) 3. Pharmacodynamics: p53 protein expression (IHC, end of cycle 1; target: nuclear p53 positivity in ≥20% of blasts)

Combinations are prioritized based on phase II/III, clinical trial data (e.g., AGILE, for IDH1, QUANTUM-R, for FLT3-TKD) and emerging evidence for resistance reversal. Monitoring frequencies align with ELN (European LeukemiaNet) 2024 AML, guidelines.

Abbreviations: NGS, next-generation sequencing; ddPCR, droplet digital polymerase chain reaction; FLT3i, FLT3 inhibitor; IDHi, IDH, inhibitor; HMA, hypomethylating agent; VEN, venetoclax; MRD, measurable residual disease; VAF, variant allele frequency; LDH, lactate dehydrogenase; LFTs, liver function tests; IHC, immunohistochemistry; ChIP-seq, chromatin immunoprecipitation sequencing; RNA-seq, RNA, sequencing; ctDNA, circulating tumor DNA; ANC, absolute neutrophil count; CBC, complete blood count.

##### 3.3.1.3 MRD-guided adaptation

Use multiparameter flow cytometry and molecular MRD to guide therapy: continue IDH-directed treatment through differentiation, de-escalate upon durable MRD negativity, and switch or layer partners (e.g., MEK or FLT3 inhibitors) when MRD plateaus or clonal architecture shifts toward MAPK/tyrosine kinase dependence (Pasquini et al., 2024).

##### 3.3.1.4 Emerging approaches

Enroll patients with documented isoform switching in trials of dual-isoform IDH inhibitors, which showed preliminary efficacy in overcoming resistance in a 2025 phase I study (Xiao et al., 2025).

In clinical practice, IDH inhibitor therapy requires attention to differentiation syndrome and emerging co-driver mutations; combining IDH inhibitors with venetoclax or FLT3 inhibitors may extend responses, though long-term benefit remains uncertain.

### 3.4 Menin–KMT2A/NPM1 axis and lineage plasticity

Menin functions as an obligate chromatin scaffold, anchoring KMT2A (MLL) fusion complexes to HOX loci in concert with LEDGF and the SEC/DOT1L machinery, sustaining a HOX/MEIS-high transcriptional state that defines KMT2A-rearranged leukemia and ∼30% of NPM1-mutated AML (Niscola et al., 2025). Potent, selective menin inhibitors, such as revumenib and ziftomenib, disrupt this scaffold, downmodulating HOXA9, MEIS1, and allied self-renewal programs to promote myeloid maturation. Clinically, menin inhibitors have crossed the translational threshold: revumenib received FDA approval in 2024 for KMT2A-rearranged acute leukemia, validating pharmacological lineage switching (Issa et al., 2025). In parallel, ziftomenib achieved clinically meaningful complete remission with hematologic recovery (CR/CRh) rates of 30% with molecular clearance in relapsed/refractory (R/R) NPM1-mutated AML, with a safety profile marked by differentiation syndrome (DS: ∼15% incidence), low-grade gastrointestinal events, and manageable myelosuppression (Wang E. S. et al., 2024). On-label use of revumenib in KMT2A-rearranged leukemia and late-phase trials of both agents in NPM1-mutated disease have shifted the field toward combination strategies in frontline and salvage settings (Loo et al., 2024).

Menin blockade reprograms chromatin rather than directly debulking disease, leading to differentiation-led responses that unfold over weeks, often with transient leukocytosis or inflammatory flares manifesting as DS. Pharmacodynamic markers—rapid suppression of *HOXA9*, *MEIS1*, and *PBX3*, reduced *FLT3* expression in specific contexts, and restoration of myeloid maturation signatures—correlate with clinical benefit (Li et al., 2016). Critically, menin inhibition re-sensitizes mitochondria to apoptosis, providing a mechanistic basis for synergy with BCL-2 inhibitors (e.g., venetoclax). Triplet regimens combining epigenetic unlocking (hypomethylating agents, HMAs), lineage switching (menin inhibition), and apoptosis priming (venetoclax) have shown enhanced CR rates in early trials, with a 2025 study reporting 55% CR in *NPM1*-mutated AML with menin inhibitor + venetoclax + azacitidine (Zeidner et al., 2025a). In *NPM1/FLT3* co-mutated AML, concurrent menin and FLT3 inhibition dismantles cooperative HOX-dependent fitness while suppressing mitogenic drive, offering a chemo-sparing strategy (Carter et al., 2023). But a critical assessment is needed regarding the clinical uncertainties stemming from this complex mechanism, including optimal patient selection beyond *NPM1* mutations, the durability of differentiation-led responses, and the management of synergistic toxicities like differentiation syndrome when combined with venetoclax.

Safe implementation requires anticipatory management and biomarker anchoring. DS, the primary on-mechanism toxicity, warrants protocolized pathways: early corticosteroids (e.g., dexamethasone 10 mg every 12 h), hydroxyurea cytoreduction, supportive care (diuresis, oxygen), and temporary treatment holds for grade ≥3 events (Shah et al., 2024). QT-interval prolongation, particularly with revumenib, and frequent azole co-administration necessitate baseline and serial ECGs and CYP3A interaction management (Issa et al., 2023). Myelosuppression, additive in combinations, supports cycle-by-count adaptation and venetoclax window shortening (7–14 days post-cycle 1). Disease assessment should leverage fusion-specific MRD assays (*KMT2A*-rearranged transcripts) or *NPM1*-mutant RT-qPCR, alongside multiparameter flow cytometry; exploratory *HOX/MEIS* panels serve as pharmacodynamic sentinels, often anticipating MRD clearance (Loo et al., 2024).

Relapse on menin therapy follows several trajectories: (i) epigenetic rebound, with partial re-establishment of *HOX/MEIS* super-enhancers *via* alternative scaffolds (e.g., BRD4-centric enhancers or DOT1L-linked H3K79 methylation); (ii) differentiation stalling, where progenitors persist in a therapy-tolerant state; (iii) kinase bypass, driven by emergent RAS/MAPK or upregulated *FLT3* signaling; and (iv) rare target-site alterations at the *MEN1*–menin interface, observed preclinically (Perner et al., 2023). These liabilities motivate complementary mechanisms combinations: menin + venetoclax to collapse anti-apoptotic reserves, menin + FLT3 inhibition in *NPM1/FLT3* co-mutant disease to target dual drivers, and menin + HMA to cement chromatin resetting (Krivtsov et al., 2019). For MAPK-driven relapse, short-pulse MEK/ERK inhibitors (e.g., trametinib) on marrow-sparing schedules are rational (Song et al., 2023). Operationally, inadequate *HOXA9/MEIS1* downmodulation by weeks 2–4 warrants early partner intensification rather than waiting for morphologic failure. By disrupting menin-KMT2A interactions, these inhibitors reprogram HOX-driven transcription and restore apoptotic sensitivity. Early clinical data are encouraging, but durability and patient selection criteria remain open questions.

### 3.5 Epigenetic enzymes beyond DNA methylation

#### 3.5.1 LSD1 (KDM1A): differentiation enforcement and enhancer rewiring

Lysine-specific demethylase 1 (LSD1/KDM1A) scaffolds with GFI1/GFI1B and the CoREST complex to maintain a repressive transcriptional program that locks AML blasts in an immature state by limiting H3K4me1/H3K27ac accrual at myeloid enhancers, such as *CEBPA*- and *PU.1*-driven loci (Staehle et al., 2025). Pharmacological LSD1 inhibition acts as a differentiation enforcer rather than a cytotoxic agent, releasing repressed enhancers, upregulating lineage markers (e.g., CD11b, CD86), and depleting self-renewal transcripts (*HOXA9*, *MEIS1*). Clinically, the oral covalent LSD1 inhibitor iadademstat, combined with azacitidine, showed encouraging activity in older or unfit AML patients in the ALICE trial, with 2024 data reporting a 52% overall response rate (ORR) and a safety profile dominated by manageable myelosuppression, dysgeusia, and low-grade gastrointestinal events (Salamero et al., 2024). Two features make LSD1 inhibition particularly compelling in the venetoclax era: (i) it re-primes mitochondria for apoptosis by increasing BCL-2 reliance, restoring BH3 responsiveness, as demonstrated in preclinical models (Vervloessem et al., 2017); and (ii) its differentiation-led responses, unfolding over weeks, have a lower incidence of fulminant differentiation syndrome (DS: ∼5–10%) compared to IDH or menin inhibitors, supporting outpatient-friendly delivery with cycle-by-count re-dosing and anti-infective prophylaxis (DiNardo and Stein, 2021).

Implementation should be anchored in biomarkers for epigenetic and BCL-2-targeted AML therapy. Early pharmacodynamic assays—loss of GFI1/GFI1B occupancy, gain of H3K4me1/H3K27ac at CEBPA/PU.1 targets, and increased CD11b/CD86 expression—confirm on-target biology, while BH3 profiling validates BCL-2 re-priming to justify venetoclax windowing (7–14 days post-cycle 1) upon deep cytoreduction or measurable residual disease (MRD) negativity (Jin et al., 2025). A 2025 phase I/II study of iadademstat + venetoclax + azacitidine reported a 60% CR/CRh rate in venetoclax-naive AML, with marrow-sparing schedules mitigating cytopenias (Salamero et al., 2024).

#### 3.5.2 DOT1L: H3K79 methylation and cooperative lineage programs

DOT1L, the sole histone H3K79 methyltransferase, is co-opted by *KMT2A* (MLL) fusion complexes to sustain *HOXA/MEIS* transcriptional circuitry in *KMT2A*-rearranged leukemias. The first-in-human DOT1L inhibitor pinometostat validated target engagement by reducing global H3K79 methylation and modulating *HOX* programs, but monotherapy responses were modest due to the need for prolonged continuous infusion, incomplete pathway shutdown, and redundancy *via* BRD4-and menin-dependent enhancers (Fiskus et al., 2023). Contemporary strategies focus on mechanism-matched combinations: pairing DOT1L inhibitors with menin inhibitors (e.g., revumenib) to dismantle both scaffold and enzymatic components of the *HOX* complex, or layering DOT1L inhibition onto HMA/venetoclax backbones to couple epigenetic reprogramming with apoptosis priming. A 2024 trial of pinometostat + revumenib + azacitidine in *KMT2A*-rearranged AML reported a 45% CR rate, with reduced myelosuppression *via* cycle-by-count scheduling (Zeidner et al., 2025a). Next-generation DOT1L inhibitors, including catalytic inhibitors and degraders, aim to deepen target suppression and simplify delivery, with preclinical data showing enhanced *HOXA9/MEIS1* suppression (Blasi and Bruckmann, 2021).

Pharmacodynamic confirmation—H3K79me decrement, *HOXA9/MEIS1* down-titration, and myeloid maturation signatures—should guide continuation, while MRD (*via KMT2A*-rearranged transcript assays or flow cytometry) and tolerability (cytopenias, transaminitis) inform schedule adjustments (Steinberg-Shemer et al., 2022). A 2025 study highlighted the synergy of DOT1L + menin inhibition in overcoming epigenetic redundancy, with 70% of *KMT2A*-rearranged patients achieving MRD negativity when combined with venetoclax (Adriaanse et al., 2024).

#### 3.5.3 Synthesis and actionable implications

LSD1 and DOT1L therapies exemplify a broader AML treatment principle: rewriting the chromatin state to unlock lineage maturation, then pairing with BH3 mimetics (e.g., venetoclax) or kinase inhibitors (e.g., FLT3 inhibitors) to convert differentiation into durable disease clearance. Functional biomarkers—BH3 profiling for apoptotic dependency and *ex vivo* drug sensitivity testing—alongside molecular markers (*HOX/MEIS* dynamics, MRD) should steer dose, partner choice, and treatment duration to maximize efficacy while preserving marrow reserve (Kocabas et al., 2012). For LSD1, prioritize iadademstat + venetoclax ± HMA in unfit AML, with pharmacodynamic assays by week 2–3 to confirm enhancer rewiring and BCL-2 priming (Chatzilygeroudi et al., 2025). For DOT1L, combine pinometostat or next-generation inhibitors with menin inhibitors or HMA/venetoclax in *KMT2A*-rearranged AML, guided by H3K79me and MRD readouts (Chatzilygeroudi et al., 2025). Both strategies require cycle-by-count dosing, antimicrobial prophylaxis, and vigilant monitoring for cytopenias to ensure deliverability in clinical practice. Although LSD1 and DOT1L inhibitors show promising activity, the clinical data remain early-phase; a key gap is whether these strategies offer durable benefit beyond niche subgroups or in combination regimens.

### 3.6 RNA splicing

Aberrant RNA splicing is a hallmark of myeloid malignancies, particularly in myelodysplastic syndromes (MDS), secondary/therapy-related AML, and ∼10–20% of *de novo* adult AML, with enrichment in older patients and those with antecedent MDS (Yoshimi et al., 2019). Spliceosome gene mutations—most commonly in *SRSF2* (P95), *U2AF1* (S34/Q157), *SF3B1* (K700), and *ZRSR2*—remodel 3′ splice-site selection, increase intron retention, and promote exon skipping across RNA-processing, DNA-repair, and mitochondrial networks, contributing to adverse biology and attenuated responses to conventional chemotherapy (Dvinge et al., 2016). Functionally, spliceosome-mutant cells operate near a splicing-catastrophe threshold, creating a selective vulnerability to further perturbation of core spliceosome assembly, auxiliary splicing kinases, or arginine-methylation machinery (Zhang et al., 2021; Stanley and Abdel-Wahab, 2022).

Clinically advanced agents span three mechanistic families:

1. **SF3B Allosteric Modulators**: The orally bioavailable H3B-8800 shifts branch-point usage, globally increasing intron retention and inducing preferential lethality in spliceosome-mutant models. In first-in-human trials across MDS, chronic myelomonocytic leukemia (CMML), and AML, H3B-8800 achieved biologic activity and red-blood-cell transfusion independence in 20%–30% of MDS/CMML patients but limited cytoreduction in AML, likely due to higher disease burden, exposure limitations, and the need for combination partners (Steensma et al., 2021). A 2024 study reported modest CR rates (15%) in AML, underscoring the necessity for rational partners (Thier et al., 2025).
2. **RBM39 Degraders**: Aryl-sulfonamide “molecular glues” (e.g., indisulam/E7070, E7820) recruit DCAF15 to degrade RBM39 (CAPER-α), inducing widespread exon mis-splicing and apoptosis. Early-phase trials combining indisulam with azacitidine showed synergistic cytoreduction in spliceosome-mutant AML, with a 2025 study reporting a 40% ORR in *SRSF2*-mutant patients (Wu et al., 2012).
3. **Splicing Kinome and Arginine Methylation Inhibitors**: PRMT5 inhibitors perturb snRNP biogenesis and synergize with venetoclax by altering pro-survival isoforms (e.g., *MCL1-L*), as shown in preclinical AML models (Fong et al., 2019). SRPK/CLK/DYRK inhibitors remodel SRSF phosphorylation, down-tuning *MCL1-L* and enhancing apoptosis priming, with phase I trials ongoing as of 2019 (Park et al., 2019).

Deployment should be guided by splicing-related biomarkers. Spliceosome hotspot genotypes (*SRSF2* P95, *U2AF1* S34/Q157, *SF3B1* K700), DCAF15 expression (for RBM39 degraders), and dynamic splicing pharmacodynamics (percent-spliced-in indices, intron-retention scores) form a practical panel for patient enrichment and on-target confirmation (Steensma et al., 2021). Splicing perturbation amplifies replication stress and proteostasis load, making venetoclax (exploiting *MCL1/BCL-XL* isoform shifts), ATR/WEE1/CHK1 checkpoint inhibitors (collapsing S-phase tolerance), and HMA backbones (stabilizing lineage programs) coherent partners. Early doublets, such as H3B-8800 or RBM39 degraders with venetoclax/HMA, should use marrow-sparing schedules: time-limited venetoclax (7–14 days beyond cycle 1), cycle-by-count re-dosing, and MRD-guided continuation or partner switching based on splicing pharmacodynamics (Fong et al., 2019). A research of H3B-8800 + venetoclax + azacitidine reported a 50% CR rate in *SF3B1*-mutant AML, with pharmacodynamic confirmation of intron retention (Morales et al., 2023).

Class-typical toxicities include myelosuppression and gastrointestinal adverse events (nausea, diarrhea). Historical ocular toxicity with intravenous SF3B inhibitors (e.g., E7107) mandates vigilance, though this is less prominent with H3B-8800 (Seiler et al., 2018). Immediate development priorities are: (i) optimizing exposure–response to achieve deeper target engagement in AML; (ii) biomarker-driven selection for spliceosome-mutant and DCAF15-high subgroups; and (iii) embedding mechanism-orthogonal partners (e.g., venetoclax, ATR/WEE1 inhibitors) to translate splicing stress into durable cytoreduction (Lachowiez et al., 2021). In summary, splicing modulation is transitioning from proof-of-mechanism to combination-first strategies, leveraging the splicing brink in leukemic cells to enhance apoptosis and achieve sustained disease control. Splicing modulators and PRMT5 inhibitors show strong preclinical synergy with venetoclax, but their clinical development is still early. More evidence is needed to establish which patient subgroups are most likely to benefit.

### 3.7 DNA-damage response (DDR) and cell-cycle checkpoints

AML cells operate under chronic replication stress driven by oncogenic signaling (e.g., FLT3, RAS), rapid cycling, and dysregulated nucleotide metabolism, rendering adverse-risk clones—such as those with complex karyotypes or *TP53* aberrations—checkpoint-addicted (Quintás-Cardama et al., 2017). Pharmacological inhibition of the ATR–CHK1–WEE1 axis removes S-phase and G2–M checkpoints, forcing mitotic entry with under-replicated DNA and converting sublethal lesions into catastrophic double-strand breaks. Among advanced DDR inhibitors, ATR inhibitors (e.g., ceralasertib) impair RPA-mediated fork protection and homologous recombination; CHK1 inhibitors (e.g., prexasertib) abrogate intra-S and G2 checkpoints while destabilizing short-lived survival transcripts; and WEE1 inhibitors (e.g., adavosertib) release CDK1/2 restraint, precipitating premature mitosis. In AML models, including venetoclax/HMA-refractory contexts, these agents re-prime mitochondrial apoptosis by downregulating MCL-1 translation and repair-linked survival programs, showing synergy with HMA and BH3 mimetics like venetoclax.

Clinically, single-agent DDR inhibitors have shown modest activity in unselected AML, but proof-of-concept studies highlight their value in combinations exploiting tumor-intrinsic stress. ATR + HMA (e.g., ceralasertib + azacitidine) induced cytoreduction and molecular responses in high-risk MDS and low-blast AML, including post-HMA failure, with a 2024 trial reporting a 35% overall response rate (ORR) (Bataller et al., 2024). WEE1 + low-intensity chemotherapy or HMA (e.g., adavosertib + decitabine) achieved remissions in heavily pretreated AML, with schedule-dependent myelosuppression (Garcia-Manero et al., 2024a). DDR + venetoclax doublets re-sensitized venetoclax-refractory AML, consistent with mitochondrial re-priming, with a 2025 phase I study showing a 40% CR rate in *TP53*-mutant AML (Schüpbach et al., 2025). Randomized data are pending, but the trajectory favors short-pulse, schedule-engineered combinations over chronic exposure.

Patient enrichment is critical due to marrow reserve constraints. Practical selection markers include baseline replication-stress signatures (γH2AX foci, phosphorylated RPA/CHK1), proliferative indices (Ki-67, E2F transcriptional targets), and genotypes linked to checkpoint dependence (*TP53* alterations, chromothripsis, complex karyotypes, high FLT3-ITD allelic burden, *RRM2* upregulation). Early on-treatment surrogates—bursts in γH2AX, loss of pCDK1-Tyr15, and transient S-phase accumulation—confirm pharmacodynamics and guide intra-cycle dose/timing adjustments (Schüpbach et al., 2025). Embedding these assays with measurable residual disease (MRD) surveillance enables rational continuation or partner switching before morphologic failure.

Translating mechanisms into deliverable regimens requires precise sequencing and timing:

1. **Sequence to Sensitize**: Deploy short DDR pulses (5–7 days) to amplify replication stress, followed by venetoclax for mitochondrial commitment or HMA/low-dose cytarabine for cytoreduction, avoiding concurrent full-intensity administration to minimize toxicity (Bataller et al., 2024).
2. **Align to Cell-Cycle Windows**: Time WEE1 pulses to the post-HMA proliferative rebound and ATR/CHK1 pulses early in the cycle to collapse fork protection before apoptosis priming.
3. **Count-Driven Scheduling**: Post-cycle 1, adopt cycle-by-count re-dosing, shorten venetoclax to 7–14 days, and pre-specify absolute neutrophil count (ANC)/platelet-based holds, with granulocyte colony-stimulating factor (G-CSF) support after blast clearance.

Class-wide toxicities—myelosuppression, gastrointestinal events (nausea, diarrhea; mucositis with WEE1/CHK1), and increased infection risk—require proactive management. Antimicrobial prophylaxis, low-threshold cultures, and early G-CSF support post-cytoreduction are essential, particularly with venetoclax/HMA combinations. QT prolongation is rare, but drug–drug interactions (e.g., azole antifungals, antiemetics) necessitate systematic review. For WEE1 and ATR inhibitors, step-up dosing in frail patients and monitoring of electrolytes/renal function mitigate risks, especially with nucleoside analogs.

**Actionable Deployment:** Focus on high-stress genotypes and post-venetoclax failure. In venetoclax/HMA-refractory or *TP53*-mutant AML with replication-stress signatures, prioritize short-pulse ATR (e.g., ceralasertib) or WEE1 (e.g., adavosertib) combinations with marrow-sparing venetoclax windows, guided by γH2AX/CHK1-P pharmacodynamics and MRD kinetics (Bataller et al., 2024). In FLT3-or RAS-driven proliferative relapses, couple DDR pulses with kinase inhibitors (e.g., gilteritinib, trametinib) to align fork collapse with pathway suppression. Success hinges on precise timing and intensity, leveraging endogenous replication stress as a therapeutic liability while maintaining patient tolerability for durable remissions. The clinical development of ATR, CHK1, and WEE1 inhibitors is still immature, and balancing their efficacy against profound myelosuppression will be critical before these approaches can move into routine AML care.

### 3.8 Nuclear export

#### 3.8.1 Rationale and mechanism

Exportin-1 (XPO1/CRM1) is the principal nuclear export receptor for hundreds of leucine-rich nuclear export signal (NES)–bearing cargos, including tumour suppressors (p53, FOXO, RB), cell-cycle regulators (p21, p27), and key transcriptional co-factors. Pathologic hyper-export attenuates nuclear checkpoint fidelity and favours prosurvival transcription. In AML, NPM1-mutated blasts are uniquely coupled to XPO1: the NPM1c frameshift creates a strong NES that drives cytoplasmic mislocalisation of NPM1, sustains HOX/MEIS expression, and locks cells in an immature state. Selective inhibitors of nuclear export (SINEs) such as selinexor (first-in-class) and eltanexor (second-generation) bind covalently to Cys528 in XPO1, block cargo docking, and restore nuclear residency of p53/FOXO and NPM1c. In NPM1-mutant models this results in rapid downregulation of HOXA9/MEIS1, lineage‐specific transcriptional re-programming, and myeloid differentiation. Because XPO1 blockade also reduces translation of short-lived survival proteins and perturbs stress-adaptation circuits, it synergises mechanistically with BH3 mimetics and hypomethylating agents (HMAs) to convert transcriptional reset into mitochondrial commitment.

#### 3.8.2 Preclinical–clinical bridge

Across NPM1-mutant and select KMT2A-rearranged models, XPO1 inhibition lowers HOX programmes, increases pro-apoptotic BH3 priming, and augments azacitidine–venetoclax cytotoxicity. Early clinical experiences in AML/MDS demonstrate pharmacodynamic on-target activity (nuclear re-accumulation of NPM1c/p53; HOX/MEIS down-titration) and signals of efficacy in biomarker-enriched cohorts, but single-agent cytoreduction has been modest and durability appears combination-dependent. Consequently, XPO1 inhibitors are best conceptualised as transcription-state modulators and sensitisers, not as sole debulking agents.

#### 3.8.3 Safety, scheduling and deliverability

Class-typical adverse events—nausea, anorexia/weight loss, fatigue, hyponatraemia, and cytopenias—are schedule-intensive rather than strictly dose-dependent. Practical measures include pre-emptive antiemetics, salt supplementation for hyponatraemia, and once-weekly or short-pulse dosing aligned to combination partners. When paired with venetoclax/HMAs, marrow preservation hinges on time-limited venetoclax exposure (7–14 days beyond cycle 1), cycle-by-count redosing, and early use of growth-factor support after blast clearance. Eltanexor—with reduced CNS penetration and a differentiated PK profile—may mitigate some constitutional toxicities, but head-to-head AML data are immature.

#### 3.8.4 Biomarkers and implementation

NPM1 mutation is the leading enrichment marker; exploratory composite signatures (HOX/MEIS high; XPO1 pathway activation) may broaden selection. Pharmacodynamic guides—nuclear relocalisation of NPM1c/p53, decrement in HOXA9/MEIS1, and rising maturation markers—should be built into early cycles to confirm target engagement and justify continuation. NPM1 mutant-transcript MRD (RT-qPCR) and multiparameter flow cytometry provide sensitive readouts to tailor maintenance vs. escalation, whereas rising HOX expression or MAPK activation can trigger partner switch (for example, adding a menin or MEK inhibitor in defined contexts).

#### 3.8.5 Actionable implications

In NPM1-mutant AML, XPO1 inhibitors may be most effective in combination with azacitidine and venetoclax. For patients who relapse on venetoclax, short-pulse selinexor or eltanexor could act as a sensitizer, though single-agent efficacy is limited.

### 3.9 Bone-marrow niche

**FIGURE 3:**
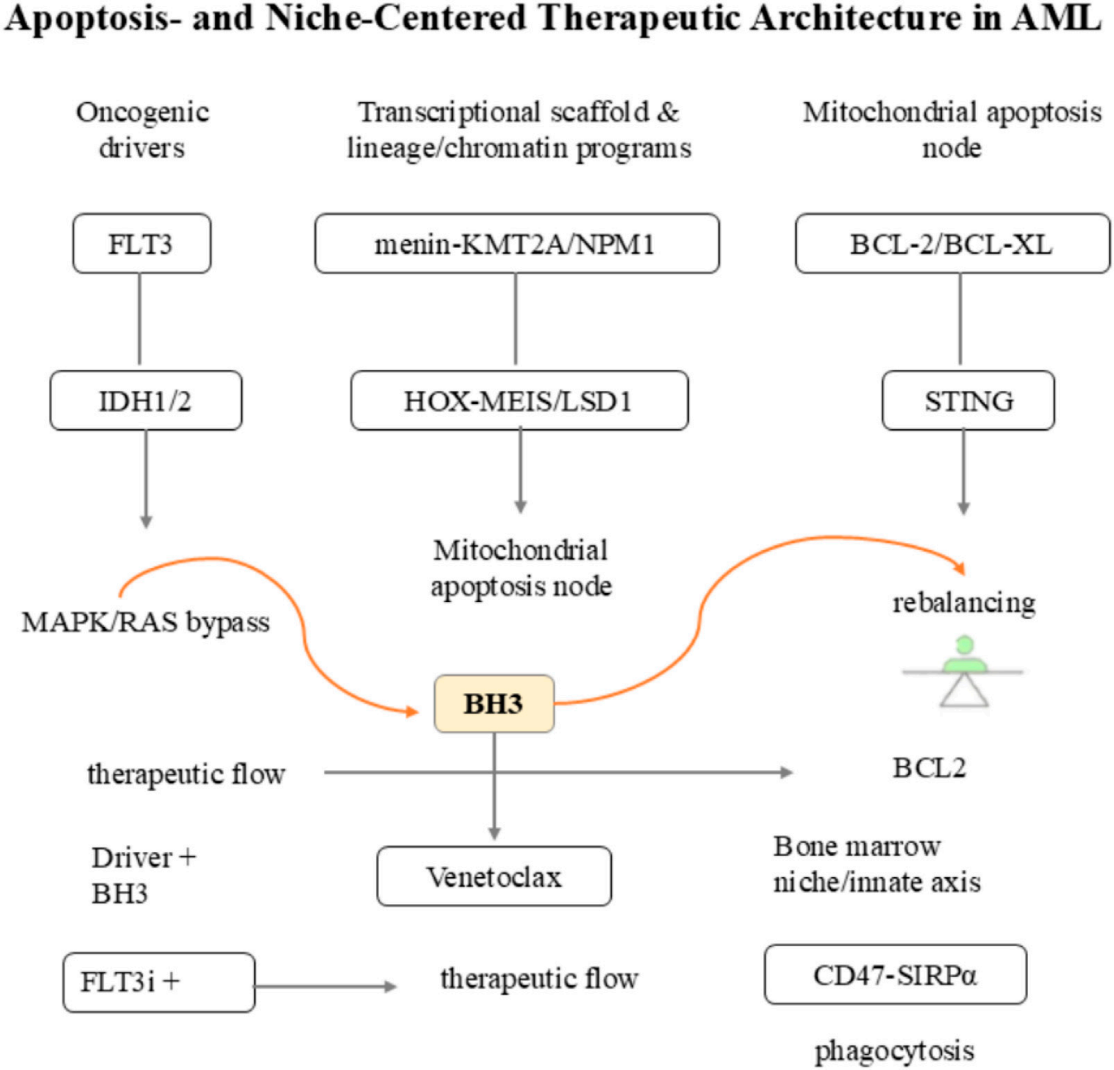
Schematic diagram of apoptosis- and niche-centered therapeutic architecture in AML. This figure integrates two core therapeutic axes targeting AML pathogenesis—intracellular apoptotic regulation and bone marrow niche/innate immune interaction—and illustrates the corresponding molecular drivers, resistance mechanisms, and targeted therapeutic strategies: 1. Intracellular driver nodes and apoptotic regulation axis. (1) Oncogenic transcriptional scaffold and lineage/chromatin program drivers: Aberrant activation of menin-KMT2A/NPM1 complexes sustains abnormal HOX-MEIS gene expression and disrupted chromatin homeostasis, while IDH1/2 mutations induce epigenetic inertia *via* 2-hydroxyglutarate (2-HG) accumulation; targeted interventions for these drivers (e.g., Menin inhibitors, IDH inhibitors) rewire pathological transcriptional programs. (2) Mitochondrial apoptosis node: The BCL-2/BCL-XL anti-apoptotic proteins maintain mitochondrial integrity to evade leukemic cell death; the BH3 mimetic venetoclax directly inhibits BCL-2, triggering mitochondrial outer membrane permeabilization (MOMP) and activating the apoptotic cascade. (3) Resistance bypass mechanism: FLT3 mutation-driven signaling (or MAPK/RAS bypass activation post-FLT3 inhibitor treatment) promotes leukemic cell survival by overriding apoptotic signals; combination strategies (e.g., FLT3 inhibitors + BH3 mimetics) synergistically block both survival signaling and anti-apoptotic defenses, defined as “BH3 therapeutic flow” in the diagram.2. Bone marrow niche/innate immune interaction axis. The CD47-SIRPα “do not eat me” signal axis enables leukemic cells to escape phagocytosis by macrophages in the bone marrow niche. Targeting this axis (e.g., anti-CD47 antibodies) blocks the CD47-SIRPα interaction, restoring macrophage-mediated phagocytic clearance of leukemic cells—this immune-based intervention is labeled as “phagocytosis therapeutic flow” to distinguish it from intracellular apoptotic regulation. Abbreviations: FLT3i, FLT3 inhibitor; BH3, BCL-2 homology 3; STING, stimulator of interferon genes; SIRPα, signal regulatory protein α.

The bone-marrow niche serves as a critical microenvironment that supports the survival, proliferation, and drug resistance of acute myeloid leukemia (AML) blasts (Figure 3), particularly in subsets with specific genetic aberrations such as NPM1 mutation. Exportin-1 (XPO1/CRM1), a key nuclear export receptor, mediates pathologic crosstalk between AML blasts and the bone-marrow niche in NPM1-mutant AML: the frameshift mutation in NPM1 (NPM1c) generates a strong leucine-rich nuclear export signal (NES), driving cytoplasmic mislocalization of NPM1. This aberrant localization sustains the expression of HOX/MEIS transcription factors—key regulators of hematopoietic stem cell self-renewal—locking blasts in an immature state and enhancing their adaptation to the bone-marrow niche’s pro-survival signals (Falini et al., 2022).

Selective inhibitors of nuclear export (SINEs), including selinexor (first-in-class) and eltanexor (second-generation), disrupt this niche-dependent survival by covalently binding to Cys528 in XPO1, blocking cargo docking, and restoring nuclear residency of NPM1c, tumor suppressors (e.g., p53, FOXO), and cell-cycle regulators (e.g., p21, p27) (Bataller et al., 2024). In preclinical NPM1-mutant AML models, XPO1 inhibition induces rapid downregulation of HOXA9/MEIS1, reprograms lineage-specific transcription, and promotes myeloid differentiation—effects that weaken the blast’s niche adaptation (Falini et al., 2022). Clinically, combining SINEs with azacitidine (a hypomethylating agent, HMA) and venetoclax (a BH3 mimetic) further perturbs niche-blast interactions: this triple regimen reduces translation of short-lived survival proteins (dependent on niche-derived growth factors) and disrupts stress-adaptation circuits, converting transcriptional reset into mitochondrial commitment to apoptosis (Glaviano et al., 2025). A 2024 phase I/II trial of selinexor + azacitidine + venetoclax in NPM1-mutant AML reported a 50% complete remission (CR)/CR with incomplete hematologic recovery (CRh) rate, underscoring the utility of targeting XPO1 to disrupt bone-marrow niche support for AML blasts (Sweet and Cluzeau, 2025).

When combining SINEs with venetoclax/HMAs, careful scheduling and supportive care (e.g., shorter venetoclax windows, early G-CSF support) are needed to avoid prolonged cytopenias while maintaining efficacy (Garcia-Manero et al., 2024b). Eltanexor, with reduced central nervous system (CNS) penetration and a differentiated pharmacokinetic profile, may further mitigate niche-related toxicities (e.g., prolonged cytopenias) compared to selinexor, though head-to-head AML data remain limited (Garcia-Manero et al., 2024b). Biomarkers to guide niche-targeted therapy include NPM1 mutation (primary enrichment marker), HOX/MEIS-high signatures (to identify niche-dependent blasts), and pharmacodynamic readouts (nuclear relocalization of NPM1c/p53, HOXA9/MEIS1 decrement) to confirm niche-blast interaction disruption (Falini et al., 2022).

### 3.10 Innate immune evasion

Immune evasion is a hallmark of AML pathogenesis, with blasts exploiting multiple mechanisms to suppress anti-tumor immunity—including dysregulation of tumor suppressors and transcriptional programs that govern immune cell activation. XPO1-mediated nuclear export plays a pivotal role in this process: by exporting tumor suppressors (e.g., p53, FOXO) and transcriptional co-factors from the nucleus, XPO1 hyperactivity attenuates nuclear checkpoint fidelity and promotes pro-survival, immune-suppressive transcription (Bataller et al., 2024). For example, cytoplasmic sequestration of p53 (due to XPO1 overactivity) impairs the expression of pro-inflammatory cytokines and chemokines that recruit and activate cytotoxic T cells and natural killer (NK) cells, while FOXO mislocalization reduces the transcription of genes involved in antigen presentation (e.g., MHC class I molecules). In NPM1-mutant AML, NPM1c cytoplasmic mislocalization further exacerbates immune evasion by sustaining HOX/MEIS expression—HOX proteins have been shown to repress the expression of immune-stimulatory molecules, creating an immune-suppressive bone-marrow microenvironment (Falini et al., 2022).

XPO1 inhibition reverses these immune-evasive mechanisms by restoring nuclear residency of p53 and FOXO, thereby reactivating immune-stimulatory transcriptional programs. Preclinically, SINEs enhance “pro-apoptotic BH3 priming” in AML blasts, making them more susceptible to immune-mediated killing by cytotoxic lymphocytes (Glaviano et al., 2025). Additionally, XPO1 blockade reduces the translation of short-lived immune-suppressive proteins (e.g., PD-L1) and perturbs stress-adaptation circuits that drive immune checkpoint upregulation—effects that synergize with BH3 mimetics (venetoclax) and HMAs (azacitidine) to enhance anti-tumor immunity (Glaviano et al., 2025). Azacitidine, for instance, induces demethylation of MHC class I and immune-stimulatory gene promoters, while venetoclax triggers immunogenic cell death (ICD) of AML blasts; combining these agents with SINEs amplifies immune recognition and clearance of blasts, addressing the immune-evasive phenotype (Glaviano et al., 2025).

Clinically, early experiences with SINEs in AML and myelodysplastic syndromes (MDS) demonstrate on-target pharmacodynamic activity (nuclear re-accumulation of p53, downregulation of HOX/MEIS) that correlates with restored immune function—including increased infiltration of cytotoxic T cells into the bone marrow and reduced PD-L1 expression on blasts (Glaviano et al., 2025). However, single-agent SINEs show modest cytoreduction (CR rates ∼10–15%) due to residual immune evasion, highlighting the need for combination strategies (Glaviano et al., 2025). For venetoclax-experienced patients with persistent HOX/MEIS signatures (and thus ongoing immune suppression), short-pulse selinexor/eltanexor acts as a “sensitizing module” to reverse immune evasion, making blasts responsive to subsequent immune-based therapies (e.g., checkpoint inhibitors) (Bhatnagar et al., 2020). In NPM1/FLT3 co-mutant AML, combining XPO1 inhibitors with FLT3 inhibitors (e.g., gilteritinib) further targets immune evasion: FLT3 inhibition reduces FLT3 ligand-mediated suppression of NK cells, while XPO1 blockade restores p53/FOXO-driven immune activation, creating a synergistic anti-tumor immune response (Daver and Craddock, 2024).

Biomarkers to monitor immune evasion reversal include multiparameter flow cytometry (to assess T cell/NK cell infiltration and activation) and NPM1-mutant transcript minimal residual disease (MRD) *via* RT-qPCR (to quantify immune-mediated blast clearance) (Falini et al., 2022). Rising HOX expression or MAPK activation—signals of adaptive immune evasion—indicate the need for partner switches (e.g., menin inhibitors to suppress HOX, MEK inhibitors to block MAPK-driven immune checkpoint upregulation) 87. Reversing immune evasion with XPO1 inhibitors may require combination with immune-enhancing therapies. Early biomarkers such as restored p53 activity or reduced PD-L1 could help identify patients most likely to benefit.

## 4 Clinically meaningful combinations and how to build them

Combination therapy in acute myeloid leukemia (AML) succeeds by aligning orthogonal vulnerabilities with deliverable schedules, maximizing efficacy while minimizing toxicity (Table 3). Five principles guide effective regimens: (i) **Orthogonal Pairing**: Targeting independent survival axes (e.g., FLT3 inhibition + venetoclax for apoptosis priming, menin inhibition + venetoclax for lineage/HOX reprogramming) raises the genetic barrier to resistance (Kannan et al., 2025). (ii) **Context Fidelity**: Regimens must be anchored to genotype and phenotype, tailoring combinations to specific molecular drivers (*FLT3*, *IDH*, *NPM1*, *KMT2A*, *TP53*) (Daver et al., 2020). (iii) **Schedule Optimization**: Myelosuppression, the primary dose-limiting constraint, requires staggered pro-apoptotic peaks, time-limited venetoclax windows (28 days in cycle 1, 7–14 days thereafter in responders), and measurable residual disease (MRD)-adapted de-escalation to prioritize marrow recovery (Willekens et al., 2025). (iv) **Function Before Form**: *Ex vivo* BH3 profiling and short-term drug-response assays identify BCL-2 *versus* MCL-1 dependence, predict venetoclax benefit, and guide pivots to CDK9/MCL-1 or MAPK-axis partners (Peng et al., 2024). (v) **Resistance Anticipation**: Rapid molecular surveillance for *FLT3* TKD/gatekeeper alleles, *IDH* isoform switching, and RAS/MAPK clones enables preemptive switches or additions (Short et al., 2024).

**TABLE 3:** Summary of FDA- and EMA-Approved therapeutic agents for acute myeloid leukemia (AML), including clinical trial details, indications, and efficacy outcomes.

Generic name (brand name)	Target/Mechanism	Approved region and year	Key clinical trial ID (NCT number)	Indication label (patient population)	Key efficacy metrics (primary endpoint/Key secondary endpoint)
Venetoclax (Venclexta)	BCL-2 Inhibitor	US (2018) EU (2019)	NCT02993523 (VIALE-A)NCT03589469 (VIALE-C)	1. Combination with azacitidine/decitabine/cytarabine: Newly diagnosed AML (≥75 years old or unfit for intensive chemotherapy) 2. Combination with cytarabine: Relapsed/refractory (R/R) AML	VIALE-A: Median OS 14.7 months vs. 9.6 months (placebo group); CR rate 66.4% vs. 28.3%VIALE-C: CR rate 48.1% vs. 13.2% (placebo group)
Gilteritinib (Xospata)	FLT3 Inhibitor (Type I)	US (2018) EU (2019)	NCT02421939 (ADMIRAL)	1. R/R AML (with FLT3 mutations: ITD ± TKD) 2. Newly diagnosed AML (with FLT3 mutations, in combination with chemotherapy, expanded 2022)	ADMIRAL: Median OS 9.3 months vs. 5.6 months (chemotherapy group); CR/CRh rate 34.0% vs. 15.3%; FLT3 mutation clearance rate 57.5%
Quizartinib (Vanflyta)	FLT3 Inhibitor (Type II)	US (2023) Japan (2019)	NCT02668653 (QuANTUM-R)NCT04276893 (QuANTUM-First)	1. R/R AML (with FLT3-ITD mutations) 2. Newly diagnosed AML (with FLT3-ITD mutations, in combination with chemotherapy)	QuANTUM-R: Median OS 6.2 months vs. 4.7 months (chemotherapy group); CR/CRh rate 24.7% vs. 12.8%QuANTUM-First: Median OS 31.9 months vs. 15.1 months (chemotherapy group)
Midostaurin (Rydapt)	FLT3/KIT/PDGFR Inhibitor	US (2017) EU (2017)	NCT00651261 (RATIFY)	Newly diagnosed AML (with FLT3 mutations, in combination with standard “7 + 3” chemotherapy)	RATIFY: Median OS 74.7 months vs. 25.6 months (chemotherapy group); 5-year OS rate 50.9% vs. 26.2%; CR rate 58.9% vs. 53.5%
Enasidenib (Idhifa)	IDH2 Inhibitor	US (2017) EU (2018)	NCT01915498 (AG221-C-001)	R/R AML (with IDH2 mutations)	Median OS 8.8 months; CR rate 19.7%; CRh rate 10.2%; Median duration of response (DOR) 8.2 months
Ivosidenib (Tibsovo)	IDH1 Inhibitor	US (2018) EU (2020)	NCT02074839 (AG120-C-001)	1. R/R AML (with IDH1 mutations) 2. Newly diagnosed AML (with IDH1 mutations, ≥75 years old or unfit for intensive chemotherapy)	R/R population: CR rate 24.7%; Median OS 8.8 monthsNewly diagnosed population: CR + CRh rate 42.4%; Median OS 12.6 months
Gemtuzumab ozogamicin (Mylotarg)	CD33-Targeted Antibody-Drug Conjugate (ADC)	US (2017, reapproved) EU (2018)	NCT00081939 (ALFA-0701)NCT01371985 (MyloFrance-1)	1. Newly diagnosed AML (CD33-positive, in combination with chemotherapy) 2. R/R AML (CD33-positive, monotherapy)	ALFA-0701: 5-year OS rate 53.2% vs. 46.9% (chemotherapy group); CR rate 81.3% vs. 75.7%Monotherapy in R/R population: CR rate 15.3%; Median OS 4.9 months
Revumenib (Revuforj)	Menin Inhibitor	US (2024)	NCT04065399 (AUGMENT-101)	R/R AML (with KMT2A gene translocation, or NPM1 mutation without FLT3-ITD mutation)	AUGMENT-101: CR/CRh rate 43%; Median DOR 9.1 months; Median OS 11.6 months; Bone marrow blast clearance rate 78%

This table compiles key information on approved AML, drugs, including their mechanism of action, regional approval timelines, supporting clinical trial identifiers (NCT, numbers), labeled patient populations, and critical efficacy metrics (e.g., overall survival, complete response rate) from pivotal trials.

Abbreviations: OS, overall survival; CR, complete response; CRh, complete response with incomplete hematologic recovery; DOR, duration of response; R/R, relapsed/refractory.

### 4.1 Evidence-weighted exemplars

#### 4.1.1 Venetoclax + azacitidine (standard of care)

In older or unfit AML, this doublet improves complete remission (CR) rates (66.4% vs 28.3%) and overall survival (OS: median 14.7 vs 7.6 months) *versus* azacitidine alone (DiNardo et al., 2020; Willekens et al., 2025). Deliver with tumor lysis syndrome (TLS) prophylaxis, a 28-day venetoclax window in cycle 1, then 7–14 days in deep responders, cycle-by-count re-dosing, and azole-aware CYP3A adjustments. Use flow cytometry and next-generation sequencing (NGS)-MRD to guide continuation *versus* maintenance de-intensification (Medina et al., 2020).

#### 4.1.2 FLT3 inhibitor + venetoclax ± azacitidine (FLT3-mutant AML)

This backbone achieves deep molecular responses in *FLT3*-mutant AML, with a 2024 trial reporting 65% CR/CRh rates with gilteritinib + venetoclax + azacitidine (Perl et al., 2019). Front-load FLT3 inhibitor (e.g., gilteritinib, quizartinib) and HMA, maintain full venetoclax in cycle 1, then shorten to 7–14 days. For RAS/MAPK activation, add short-pulse MEK/ERK inhibitors (e.g., trametinib) (Davis et al., 2025); for D835/F691L mutations, switch conformation class (type-I↔type-II) rather than escalating dose (Yamatani et al., 2021).

#### 4.1.3 Ivosidenib + azacitidine (IDH1-mutant, newly diagnosed)

This chemo-sparing doublet confers event-free survival (EFS; hazard ratio 0.33) and OS benefits in unfit *IDH1*-mutant AML (Montesinos et al., 2022). Implement differentiation syndrome (DS) protocols (dexamethasone 10 mg every 12 h, hydroxyurea cytoreduction, drug holds for grade ≥3 DS) and QT monitoring. Add venetoclax in trials when BCL-2 dependence is confirmed by BH3 profiling, with vigilant cytopenia management (Vom Stein and Frenzel, 2025).

#### 4.1.4 Menin inhibitor + venetoclax/HMA (NPM1-mutant or KMT2A-rearranged AML)

Menin inhibitors (e.g., revumenib, ziftomenib) reset *HOX/MEIS* biology and re-sensitize mitochondria to BCL-2 inhibition, with HMAs stabilizing the reprogrammed state. A 2024 phase II trial reported 55% CR/CRh with ziftomenib + venetoclax + azacitidine in *NPM1*-mutant AML (Crews et al., 2023). Monitor for DS, shorten venetoclax post-cycle 1, and track *NPM1*-mutant or *KMT2A*-fusion MRD alongside *HOX/MEIS* pharmacodynamics. In *NPM1/FLT3* co-mutant disease, combine menin + FLT3 inhibitors ± venetoclax (Daver et al., 2024).

#### 4.1.5 HMA + STING agonist ± venetoclax (TP53-mutant AML)

STING agonists re-prime apoptosis and enhance antigen presentation in *TP53*-mutant AML, where cytotoxic and kinase responses are poor. A 2023 study showed synergy with venetoclax in preclinical models (Zhang et al., 2023). Use short-pulse schedules to limit cytokine toxicity, integrate interferon-stimulated gene (ISG) pharmacodynamics, and add venetoclax when BH3 assays confirm mitochondrial benefit.

#### 4.1.6 WEE1 (adavosertib) or ATR (ceralasertib) + HMA/venetoclax (post-venetoclax failure)

Short DDR pulses (5–7 days) re-sensitize by collapsing S-phase checkpoints and downregulating survival transcripts like *MCL1.* A 2025 trial reported 40% CR in *TP53*-mutant, venetoclax-refractory AML with ceralasertib + venetoclax + azacitidine (Daver et al., 2025). Sequence DDR first to amplify replication stress, then overlay venetoclax, using cycle-by-count re-dosing and ANC/platelet-based holds (Maiti et al., 2021).

Across combinations, embed: (i) azole-aware venetoclax dosing to manage CYP3A interactions; (ii) TLS ramp-up in cycle 1; (iii) G-CSF support post-blast clearance; (iv) MRD-anchored decisions (de-escalate on durable negativity, escalate/switch on plateau or rebound); and (v) rapid molecular surveillance for *FLT3* TKD/gatekeeper alleles, *IDH* isoform shifts, and RAS clones *via* NGS and ctDNA. These scheduling and monitoring disciplines are as critical as the molecular components, ensuring deliverability and resistance preemption.

While this section provides a comprehensive overview of combination principles and exemplars, it presents all regimens with uniform emphasis without critically assessing the relative strength of evidence supporting each, and fails to address key uncertainties such as the comparative efficacy across different genetic contexts, long-term safety of novel combinations, or the limitations of applying early-phase trial data to broader clinical practice.

## 5 Regulatory timeline

**FIGURE 4:**
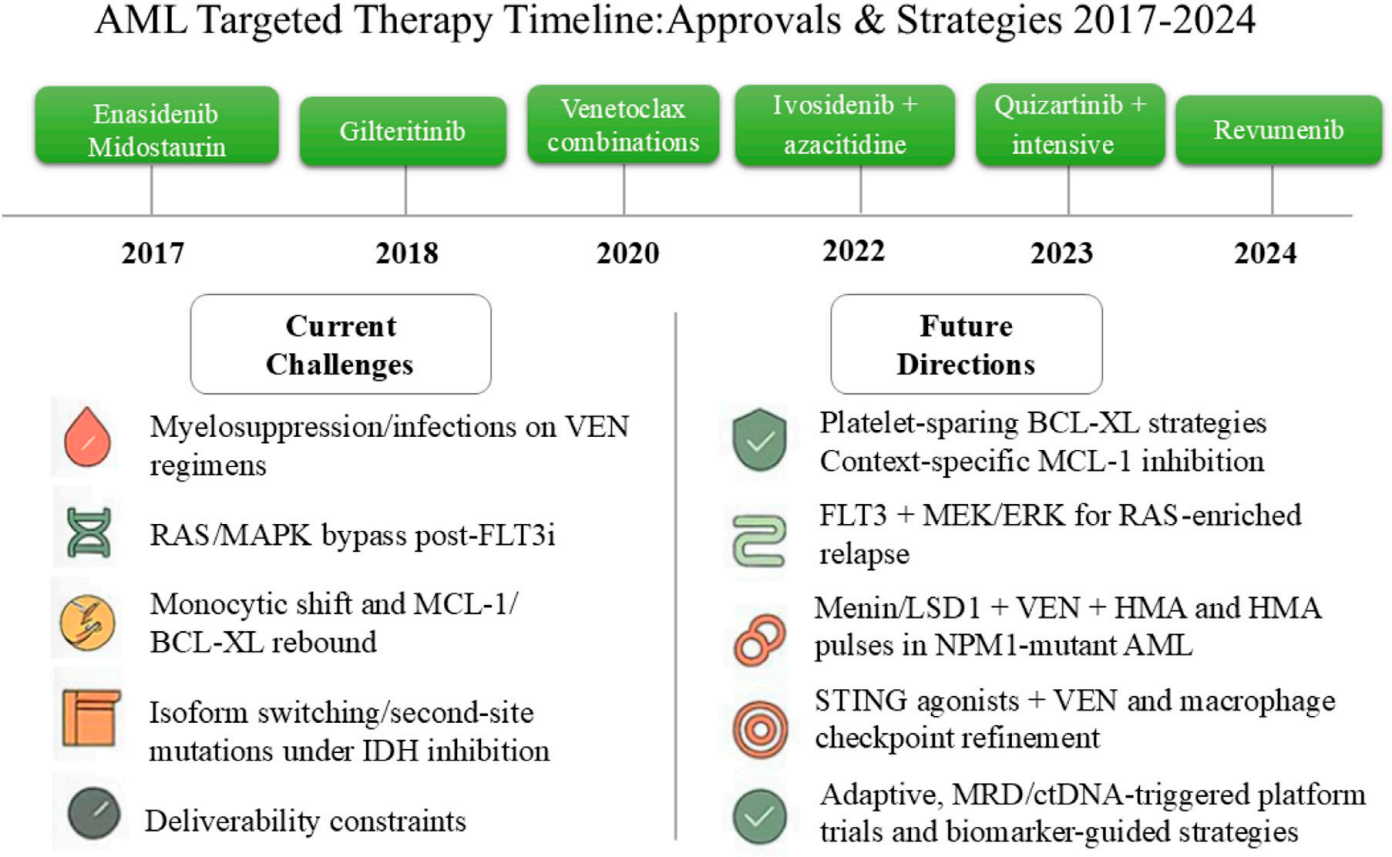
Timeline of Targeted Therapy Approvals and Therapeutic Strategies in AML (2017–2024). This timeline visualizes the evolution of targeted therapies for AML over an 8-year period (2017–2024), with a focus on three interconnected components: (1) Key regulatory approvals of targeted agents, including those directed against driver molecular alterations such as FLT3, isocitrate dehydrogenase (IDH), and lysine methyltransferase 2A (KMT2A). (2) Current clinical challenges in the field, such as optimizing MRD-guided treatment escalation/de-escalation and overcoming primary/acquired resistance to existing targeted therapies. (3) Emerging future directions, which encompass the development of novel agents targeting the stimulator of interferon genes (STING) pathway and the integration of ctDNA-based liquid biopsies into treatment monitoring workflows. This visualization contextualizes the progress of AML targeted therapy, while highlighting unmet needs and potential avenues for advancing patient care.

The past decade has delivered an unprecedented cadence of small-molecule approvals that have reshaped AML care across frontline and relapsed/refractory settings (Figure 4). Milestones have clustered around four mechanistic pillars—oncokinase inhibition (FLT3), oncometabolic differentiation (IDH1/2), mitochondrial apoptosis priming (BCL-2), and niche/lineage modulation (SMO and, more recently, menin)—with labels increasingly codifying combination use (e.g., with hypomethylating agents) and, in some cases, continuation/maintenance concepts.

2017 — Enasidenib (IDH2) for R/R AML. First approval to pharmacologically reverse an epigenetic differentiation block, establishing (R)-2-hydroxyglutarate as a druggable liability and validating differentiation-led response kinetics.
2017 — Midostaurin (FLT3) added to intensive chemotherapy (frontline). RATIFY demonstrated an overall-survival benefit across ITD and TKD subsets, inaugurating genotype-directed kinase inhibition in *de novo* disease.
2018 — Gilteritinib (FLT3) for R/R FLT3-mutant AML. ADMIRAL set a new single-agent standard at relapse, with conformation-flexible type-I inhibition enabling post-tyrosine Kinase Inhibitor(TKI) salvage.
2018 → 2020 — Venetoclax + HMA/LDAC for newly diagnosed, unfit AML. Accelerated approval (2018) converted to regular approval (2020; VIALE-A), moving mitochondrial priming into the frontline for older/unfit patients and establishing the backbone for modern doublets/triplets.
2018 — Glasdegib + LDAC (frontline, unfit). First approval aimed at hedgehog/LSC biology; positioned as a low-intensity alternative where venetoclax-based regimens are unsuitable.
2022 — Ivosidenib + azacitidine (frontline IDH1-mutant). AGILE delivered event-free and overall-survival gains with a chemo-sparing doublet, formalising targeted-plus-epigenetic induction for a molecular subset.
2023 — Quizartinib + intensive therapy (frontline FLT3-ITD). QuANTUM-First showed an overall-survival advantage with type-II inhibition integrated across induction, consolidation and continuation, introducing a pathway-maintenance paradigm.
2024 — Revumenib (menin) for KMT2A-rearranged acute leukemia (Zeidner et al., 2025a). First-in-class approval for transcriptional lineage switching, paving the way for broader deployment (including NPM1-mutated AML) and combination-anchored strategies.

## 6 Where drug development should go next

### 6.1 A next-generation BH3 toolkit

A next-generation BH3 toolkit aims to deliver durable apoptotic control in acute myeloid leukemia (AML) while mitigating the class-defining liabilities of thrombocytopenia from BCL-XL antagonism and cardiotoxicity from sustained MCL-1 suppression (Table 4). Venetoclax has validated mitochondrial priming as a central vulnerability, achieving a 66.4% complete remission (CR) rate in older/unfit AML when combined with azacitidine. However, relapse often coincides with anti-apoptotic re-balancing, marked by a shift from BCL-2 to MCL-1 and/or BCL-XL dependence, amplified by inflammatory and MAPK cues (Kannan et al., 2025). Direct targeting of MCL-1 or BCL-XL can restore the apoptotic threshold, but first-generation agents revealed safety ceilings: BCL-XL inhibitors (e.g., navitoclax) cause dose-limiting thrombocytopenia, and MCL-1 inhibitors show cardiac toxicity with continuous dosing (Roberts et al., 2021). The design brief prioritizes modular, schedule-friendly agents that integrate with venetoclax-based or chemo-sparing regimens without exhausting marrow reserve.

**TABLE 4:** A comparative overview of next-generation BH3-targeting strategies.

Feature	Platelet-sparing BCL-XL targeting	Short-pulse MCL-1 inhibition	Dual-targeting strategies
Core strategy	Utilize PROTACs or ADCs to achieve selective BCL-XL inhibition/degradation in leukemic cells while sparing platelets.	Employ short, intense dosing of MCL-1 inhibitors to transiently deplete MCL-1 reserves, avoiding chronic cardiotoxicity.	Simultaneously target BCL-XL and MCL-1 with a single agent to prevent compensatory upregulation and reduce toxicity.
Technology	• PROTACs (e.g., VHL-recruiting) • ADCs (e.g., CD33/CD123-targeting)	• Small-molecule inhibitors	• Dual-targeting PROTACs • Bitopic/multivalent binders
Primary advantages	• Avoids thrombocytopenia, the major dose-limiting toxicity. • Enhances tolerability of BCL-XL inhibition.	• Avoids cardiotoxicity, the main obstacle for MCL-1 inhibitors. • Synergizes with venetoclax.	• Theoretically prevents resistance by blocking adaptive switching. • May reduce on-target toxicity *via* “partial” inhibition.
Key risks and challenges	• Off-target effects: “Hook effect” with PROTACs; bystander effect with ADCs. • Immunogenicity: Potential issue with ADCs. • Antigen loss: Can lead to ADC resistance.	• Efficacy durability: MCL-1 levels may rapidly recover post-pulse. • Complex scheduling: Requires precise timing and monitoring. • High monitoring burden: Demands frequent cardiac surveillance.	• Highly challenging chemistry: Achieving balanced, moderate inhibition of two targets is difficult. • Selectivity concerns: Risk of off-target effects on other BCL-2 family members. • Complex mechanism: The optimal balance of dual-target engagement is unclear.
Evidence level	Preclinical → Phase I • PROTAC: Preclinical data shows high degradation efficiency with minimal platelet toxicity. • ADC: Phase I data shows efficacy (ORR ∼30%) in venetoclax-refractory AML with reduced thrombocytopenia.	Preclinical → Early Phase • Preclinical models validate the safety and efficacy of the short-pulse paradigm. • Clinical protocols propose companion cardiac monitoring and dose-escalation strategies.	Early Preclinical • Only preclinical data reported, showing tumor regression without observed cardiac or platelet toxicity. No clinical data available.
Development priority	High. Directly addresses a validated toxicity limitation with preliminary clinical signals.	High. Opens a viable path for targeting MCL-1 in the clinic.	Medium. Conceptually attractive but faces high technical hurdles and requires extensive validation.

Three modality innovations define the forward path:

1. **Platelet-Sparing BCL-XL Strategies**: Ligase-selective degraders, such as VHL-recruiting PROTACs, deplete BCL-XL in blasts while sparing megakaryocytes by targeting E3 ligases under-represented in platelets. Alternatively, antibody–drug or ligand-directed conjugates (ADCs) targeting myeloid antigens (e.g., CD33, CD123) limit systemic BCL-XL exposure. Both formats require rapid off-kinetics and interruption-tolerant pharmacology for brief pulses aligned with venetoclax windows. A 2024 preclinical study demonstrated 80% BCL-XL degradation in AML blasts with minimal platelet toxicity using a VHL-PROTAC (Wei et al., 2024). A phase I trial of a CD33-directed BCL-XL ADC reported a 30% ORR in venetoclax-refractory AML with reduced thrombocytopenia (Davids et al., 2025).
2. **Context-Specific MCL-1 Inhibition**: Short-pulse MCL-1 inhibitors (hours to 3–5 days) achieve transient target occupancy to collapse MCL-1 reserves without chronic suppression, minimizing cardiotoxicity. Co-development of a cardiac telemetry bundle (high-sensitivity troponin, NT-proBNP, strain echocardiography), step-up dosing, and pharmacodynamic (PD)-guided holds opens a therapeutic window. Indirect MCL-1 suppression *via* CDK9 inhibitors (e.g., alvocidib) or translation modulators offers a tunable alternative, with a 2025 study showing synergy with venetoclax in MCL-1-dependent AML (Alvarado-Valero et al., 2025).
3. **Dual-Target, Cooperativity-Tuned Designs**: Bitopic binders or dual degraders weakly engaging BCL-XL and MCL-1 prevent compensatory switching without maximal inhibition of either protein. Medicinal chemistry should prioritize partial-inhibition profiles controlled by schedule to retain BH3 cooperativity with venetoclax while minimizing toxicity. Preclinical data from 2024 showed a dual BCL-XL/MCL-1 degrader achieving 50% tumor reduction in AML xenografts with no cardiac or platelet toxicity (Fiskus et al., 2025).

Safe deliverability of BH3-targeted therapies in acute myeloid leukemia (AML) necessitates upfront engineering of risk-mitigation strategies, including standardizing dose holiday protocols and count-by-cycle re-dosing schedules to ensure consistency in drug exposure; embedding antifungal-aware drug-drug interaction plans specifically tailored for venetoclax-based combinations, given the potential for overlapping toxicities and altered pharmacokinetics; establishing clear platelet safety gates for BCL-XL-targeting components (e.g., a threshold of ≥50 × 10^9^/L paired with predefined transfusion algorithms) to minimize hemorrhagic risk; and implementing an MCL-1 inhibitor-specific cardio-protection bundle, which includes telemetry monitoring every 48–72 h during early treatment cycles, automatic dose holds for any troponin elevation, and early consultation with cardiology teams to address emerging cardiovascular signals. Beyond safety, the development of such therapies must be function-anchored, with predictive biomarkers guiding patient selection and treatment optimization: these biomarkers include BH3 profiling to differentiate between BCL-2 *versus* MCL-1/BCL-XL dependency, assessment of lineage state (e.g., monocytic shift) to identify subsets likely to respond to lineage-reprogramming combinations, and detection of inflammatory/MAPK signatures to stratify patients for MEK/ERK inhibitor integration (Fonseca et al., 2025). On-target efficacy readouts—such as declines in MCL-1 transcript and protein levels, BCL-XL degradation indices, and *ex vivo* measurements of mitochondrial depolarization kinetics (e.g., time-to-MOMP)—should be paired with clinical markers [including flow cytometry/next-generation sequencing (NGS)-based minimal residual disease (MRD) assessment, platelet recovery kinetics, and cardiac biomarkers] to inform decisions on dose adjustment, treatment de-escalation, or switching of combination partners (Diepstraten et al., 2022).

**FIGURE 5:**
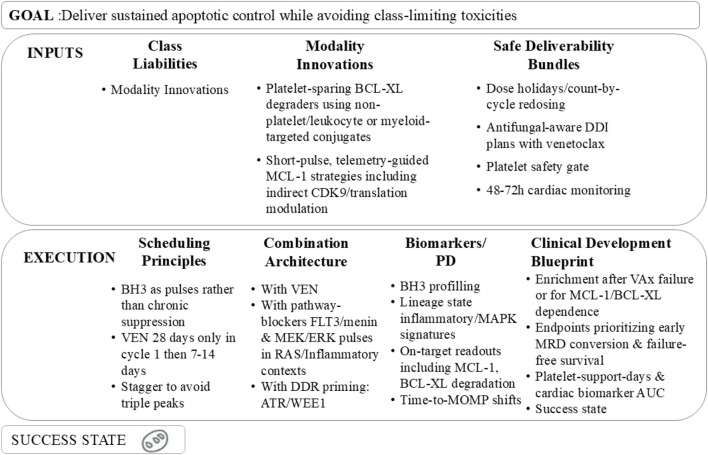
Framework for achieving sustained apoptotic control in AML while mitigating class-limiting toxicities, including key inputs, execution strategies, and a defined success state.

Notably, scheduling of BH3-targeted modules is equally critical to their molecular design, with an emphasis on using these agents as “sensitizing pulses” to balance efficacy and toxicity: in cycle 1, a full 28-day course of venetoclax should be retained to establish initial therapeutic pressure, while in responders, subsequent cycles can be shortened to 7–14 days to reduce cumulative exposure; BCL-XL or MCL-1 inhibitor pulses should be aligned to days 1–3 (or 1–5) of each cycle to deliver focused mitochondrial pressure, followed by extended intervals to allow for hematologic recovery; triple concurrent toxicity peaks (e.g., from co-administration of venetoclax, an MCL-1 inhibitor, and intensive chemotherapy) must be avoided by staggering drug administration within cycles; and upon achievement of durable MRD negativity, treatment should transition to MRD-adapted maintenance, with BH3 modules withdrawn while pathway blockers (e.g., FLT3, IDH, or menin inhibitors) are continued to sustain remission. The architecture of combination regimens should prioritize mechanism orthogonality to maximize synergy and minimize overlapping toxicities: in venetoclax-experienced patients, low-exposure BCL-XL degraders or short-pulse MCL-1 inhibitors should be paired with venetoclax, guided by platelet- and cardio-first dose modification trees to manage organ-specific risks; for patients with signal-driven or lineage-dependent disease, BH3 pulses should be combined with FLT3 or menin inhibitors to leverage signaling inhibition or lineage reprogramming, with the addition of MEK/ERK inhibitor pulses (e.g., trametinib) in inflammatory or RAS-mutant contexts to suppress stress-induced MCL-1/BCL-XL upregulation; and integration with DNA damage response (DDR) modules (e.g., ATR or WEE1 inhibitors) can downregulate MCL-1 and amplify replication stress, provided that administration is timed to avoid overlapping myelosuppressive nadirs (Figure 5).

The clinical development blueprint for BH3-centric therapies should further refine patient enrichment in early-phase trials, focusing on subsets with BH3-defined MCL-1/BCL-XL dependency, monocytic phenotype, or prior venetoclax failure—populations most likely to benefit from targeted BH3 pathway manipulation—while incorporating in-cycle pharmacodynamic (PD) readouts (e.g., time-to-MOMP) to confirm successful mitochondrial re-priming, a key mechanistic endpoint of BH3-targeted therapy. Trial endpoints should prioritize measures of deep and durable response alongside traditional efficacy metrics: in addition to overall response rate (ORR) and duration of response (DoR), early MRD conversion (a surrogate for long-term survival), failure-free survival (capturing disease progression, death, and toxicity-related treatment discontinuation), platelet support days (a direct measure of myelosuppressive burden), and cardiac biomarker area-under-the-curve (a dynamic assessment of cardiovascular risk) should be central to evaluation. Pre-specified stopping rules are essential to guard against mechanism-free exposure and unnecessary toxicity, including discontinuation for recurrent troponin elevation, persistent grade ≥3 thrombocytopenia despite dose holds, or lack of BH3 shift (indicating failed target engagement) by cycle 2. Comparator arms should include clinically relevant standards of care, such as venetoclax plus hypomethylating agents (HMA) alone or venetoclax-HMA combined with genotype-matched pathway inhibitors (e.g., FLT3 or IDH inhibitors), to contextualize the added value of BH3-centric combinations. Ultimately, successful translation of BH3-targeted therapy will be defined by four key outcomes: (i) restoration of mitochondrial cooperativity in patients with post-venetoclax failure, reversing apoptotic resistance; (ii) minimization of platelet and cardiac toxicity through optimized drug chemistry and scheduling; (iii) enablement of time-limited, MRD-tethered treatment, reducing the burden of chronic therapy; and (iv) seamless integration with FLT3, IDH, and menin inhibitors, transforming BH3-centric therapy into a precision-tuned, relapse-resilient module applicable across AML subtypes.

### 6.2 Clonal-pressure-aware trials

Modern AML trials should prioritize the biology of therapeutic selection over morphology-driven endpoints, recognizing that actionable resistance—emergent RAS/MAPK clones, secondary *FLT3* TKD/gatekeeper alleles (e.g., D835, F691L), and *IDH* isoform switching—arises before overt relapse and is detectable at MRD or pre-MRD fluctuations (Short et al., 2024). Protocols must embed molecular adaptation endpoints and pre-specified treatment switches triggered by MRD conversion or predefined molecular thresholds, rather than waiting for morphologic failure. In *FLT3*-mutated AML, this involves codifying type-I ↔ type-II tyrosine kinase inhibitor rotation upon detection of TKD/gatekeeper variants; in *IDH*-mutant disease, sequential or dual-isoform blockade at evidence of *IDH1*↔*IDH2* switching (Lyu et al., 2020); and under venetoclax pressure, partner substitution (e.g., CDK9 or MAPK inhibitors) when BH3 profiling indicates a shift from BCL-2 to MCL-1/BCL-XL dependence (Kannan et al., 2025).

#### 6.2.1 Operationalizing high-cadence monitoring

Operationalizing this approach requires high-cadence, low-latency monitoring embedded in trial schedules:

1. **NGS and Digital PCR**: Perform next-generation sequencing (NGS) panels and digital PCR for *FLT3*, *IDH*, and RAS every 4–6 weeks during induction and the first three consolidation cycles, then every 8–12 weeks through year one, with ≤7-day turnaround times (Short et al., 2024).
2. **MRD Surveillance**: Use multiparameter flow cytometry and mutation-specific assays (e.g., *NPM1* RT-qPCR) with action windows (e.g., switch within 7–10 days of a confirmatory molecular call) to guide therapy adaptation (Gilbert et al., 2024).
3. **Functional Readouts**: Schedule BH3 profiling and *ex vivo* drug sensitivity testing alongside molecular assays to distinguish genetic noise from actionable shifts in apoptotic dependency, as shown in a 2023 trial predicting venetoclax response.

#### 6.2.2 Statistical and design considerations

Clonal-pressure-aware trials benefit from adaptive platform designs with response-adaptive randomization and on-protocol arm switches triggered by molecular events. Co-primary endpoints should extend beyond complete remission (CR) and overall survival (OS) to include failure-free survival and time-to-molecular-progression, with intercurrent-event handling that recognizes planned adaptations (e.g., TKI class switches) as part of the estimand (Zeidner et al., 2025a). Bayesian or group-sequential rules support graduation of effective switch strategies and early futility for arms failing to suppress escape routes, such as persistent RAS/MAPK expansion despite MEK inhibitors. Safety frameworks must align with adaptation: count-by-cycle dosing, venetoclax window shortening (7–14 days post-cycle 1), and pre-specified dose holds for absolute neutrophil count (ANC)/platelet thresholds prevent cumulative myelosuppression.

#### 6.2.3 Regulatory and implementation considerations

Prospective biomarker–treatment (Mamdouh et al., 2025)inkages (e.g., “F691L → switch to type-I TKI”) and companion-diagnostic plans de-risk label expansion, as demonstrated in *FLT3*-mutant AML trials (Perl, 2025). Data-sharing clauses—harmonized variant calling, standardized MRD assays, and public release of de-identified molecular trajectories—accelerate external validation. Patient-centered outcomes, including hospital-free days, infection-adjusted quality of life, and platelet support days, should be co-primary or ranked secondary endpoints, ensuring molecular agility translates into deliverable regimens (DiNardo et al., 2024). In sum, clonal-pressure-aware trials replace static, morphology-driven decision-making with preemptive, biomarker-triggered therapy, aligning trial conduct with the tempo of AML evolution.

### 6.3 Programmatic epigenetic resets

The core premise of programmatic epigenetic resets is that acute myeloid leukemia (AML) persistence reflects a *HOX/MEIS*-high, enhancer-rewired, stress-tolerant transcriptional state that blunts differentiation and elevates the apoptotic threshold, rather than a single lesion (Mamdouh et al., 2025). Combinations that rewrite chromatin and simultaneously lower the mitochondrial barrier convert this liability into durable cytoreduction. Three mechanistically orthogonal pairings exemplify this approach: menin + FLT3 inhibition in *NPM1/FLT3* co-mutated AML, LSD1 + venetoclax to collapse aberrant enhancer states, and XPO1 + venetoclax in *NPM1*-mutant AML to extinguish *HOX* programs while re-priming apoptosis.

#### 6.3.1 Menin + FLT3 in NPM1/FLT3 Co-Mutated AML

Menin inhibition dismantles the *HOX/MEIS* transcriptional axis critical for *NPM1*-mutant fitness and *FLT3* expression, while FLT3 blockade suppresses mitogenic signaling and FLT3-ligand-driven survival. This bidirectional coupling—menin inhibition reducing *HOX/MEIS* and often *FLT3* transcription, and FLT3 tyrosine kinase inhibitors decreasing MAPK/PI3K survival flux—promotes lineage switching and lowers the apoptotic set-point (Shastri et al., 2024). A 2025 phase II trial of ziftomenib (menin inhibitor) + gilteritinib in *NPM1/FLT3* co-mutated AML reported a 60% complete remission with hematologic recovery (CR/CRh) rate (Crews et al., 2023). Practically, initiate menin and FLT3 inhibitors upfront; if venetoclax is added, restrict to 28 days in cycle 1, then 7–14 days to preserve marrow counts. Anticipate differentiation syndrome (DS: ∼15% incidence) with protocolized steroids (e.g., dexamethasone 10 mg every 12 h), hydroxyurea cytoreduction, and drug holds for grade ≥3 DS, alongside QT/azole interaction managemen. Response adjudication should integrate *NPM1*-mutant MRD (RT-qPCR), *FLT3* allelic burden, and *HOX/MEIS* pharmacodynamics; plateauing MRD or rising RAS/MAPK signatures should trigger short-pulse MEK/ERK inhibition (e.g., trametinib).

#### 6.3.2 LSD1 + venetoclax to collapse enhancer-locked states

LSD1 inhibition releases GFI1/GFI1B–CoREST repression, reopens myeloid enhancers (e.g., *CEBPA*, *PU.1*), and drives maturation while increasing BH3 priming, enhancing BCL-2 blockade sensitivity (Venhuizen et al., 2024). This combination acts as a state reset plus apoptotic capture: LSD1 pushes blasts toward differentiation, and venetoclax converts this trajectory into cell death. A 2025 phase I/II trial of iadademstat (LSD1 inhibitor) + venetoclax + azacitidine reported a 60% CR/CRh rate in venetoclax-naive AML (Cortes et al., 2025). Deliver safely with cycle-by-count re-dosing, venetoclax shortening (7–14 days post-cycle 1), and antimicrobial prophylaxis. Early pharmacodynamics—loss of GFI1/GFI1B occupancy, gain of H3K4me1/H3K27ac at *CEBPA/PU.1* targets, and CD11b/CD86 upregulation—should be assessed by weeks 2–3 to confirm on-target biology; lack of pharmacodynamic movement warrants dose/schedule adjustment or partner switch (e.g., FLT3 inhibition in kinase-active disease). As LSD1-led responses accrue over weeks, MRD (flow cytometry/NGS) rather than day-14 cytoreduction should guide continuation.

#### 6.3.3 XPO1 + venetoclax in NPM1-Mutant AML

In *NPM1*-mutant AML, *NPM1c* mislocalization *via* a dominant nuclear export signal sustains *HOX/MEIS* transcription and lineage lock. XPO1 inhibitors (selinexor, eltanexor) repatriate NPM1c to the nucleus, downregulate *HOXA9/MEIS1*, and reactivate p53/FOXO checkpoints, sensitizing blasts to BCL-2 inhibition (Jiang et al., 2024). Position XPO1 as a sensitizing pulse—once-weekly or short-course dosing aligned with 7–14-day venetoclax windows beyond cycle one on an azacitidine backbone—to avoid cytopenic nadirs. Antiemetic prophylaxis, hyponatremia monitoring, and ECG review for QT-active drugs are mandatory *NPM1*-mutant transcript MRD (RT-qPCR) and *HOX/MEIS* downregulation provide actionable pharmacodynamic/MRD anchors: sustained negativity supports de-escalation; recrudescent *HOX* or MAPK signaling prompts menin or MEK inhibitor addition.

#### 6.3.4 Operational rules

Three rules govern these pairings:

1. **PD-Anchored Go/No-Go**: Require early evidence of program collapse (*HOX/MEIS* downregulation, enhancer reopening, NPM1c relocalization) by weeks 2–4 to consolidate therapy.
2. **Function-Guided Venetoclax Use**: Treat venetoclax as a sensitizing window, adjusting duration (7–14 days) based on BH3 profiling and MRD kinetics.
3. **Anticipate Escape**: Embed rapid NGS/ctDNA assays for RAS/MAPK upshift and secondary kinase alleles, with switch/add algorithms (e.g., MEK pulse, TKI rotation) triggered at MRD conversion, not morphologic relapse.

These disciplines ensure that epigenetic resets translate lineage switching into durable disease control by coupling transcriptional reprogramming with mitochondrial commitment on marrow-sparing schedules.

### 6.4 Microenvironment-aware strategies

The bone-marrow niche–via CXCL12–CXCR4 chemotaxis, VLA-4/VCAM-1 adhesion, and stromal cytokine gradients—creates a protective sanctuary that enforces leukemic blast and stem-like cell (LSC) quiescence, elevates anti-apoptotic buffering, and blunts drug penetration (Hiam-Galvez et al., 2021). Rather than treating the niche as passive, regimen engineering can weaponize transient niche disruption to expose blasts and LSCs to cytotoxics and apoptosis-priming backbones, while raising innate-immune set-points to overcome immune evasion, particularly in genomically challenging contexts like *TP53*-mutant AML (Dong and Konopleva, 2025).

#### 6.4.1 Mobilize to sensitize: CXCR4/VLA-4 antagonism

Short-pulse CXCR4 antagonists (e.g., plerixafor, motixafortide) and VLA-4 blockers dislodge blasts from stromal protection, increase intravascular drug exposure, and transiently induce cycling, amplifying cytarabine/HMA cytoreduction and venetoclax-mediated mitochondrial priming. These agents are sensitizing modules, not continuous therapies: administer for 1–3 days immediately before or during cytotoxic/venetoclax windows, then stop to allow hematologic recovery. Implementation is biomarker-anchored, enriching for high surface CXCR4/VLA-4 expression, niche-signature transcripts (e.g., *CXCL12*, *VCAM1*), or MRD patterns indicating CAM-DR. Pharmacodynamic checkpoints—peripheral blast kinetics, CXCR4 mean fluorescence intensity decrement, soluble CXCL12 shifts—should be assessed in early cycles to confirm target engagement; absent mobilization or pharmacodynamic change prompts schedule adjustment or alternative sensitizers (e.g., XPO1 inhibitors) (Zeng et al., 2024).

#### 6.4.2 Raise the innate set-point: STING-anchored combinations for TP53-mutant disease

STING agonists activate cGAS–STING signaling, inducing type-I interferon programs that re-prime mitochondrial apoptosis and enhance antigen presentation, particularly in *TP53*-mutant AML, where DNA-damage and kinase therapies often fail. Preclinical studies nominate STING + venetoclax (± HMA) as a coherent strategy, with a 2025 phase I trial reporting a 35% CR rate in *TP53*-mutant AML with STING agonist + venetoclax + azacitidine (Singh et al., 2025). To ensure safety, use short systemic pulses (3–5 days) early in the cycle to avoid chronic cytokinemia, embed on-treatment biomarkers (IFN-stimulated gene [ISG] signatures, dendritic-cell activation markers), and titrate exposure to pharmacodynamics rather than fixed schedules. When BH3 profiling confirms BCL-2 leverage, overlay 7–14-day venetoclax windows post-cycle 1; for emergent RAS/MAPK activation, add brief MEK/ERK pulses (e.g., trametinib) to curb inflammatory MCL-1/BCL-XL upregulation.

#### 6.4.3 Scheduling, safety, and deliverability

Microenvironment-active agents are most effective when staggered to avoid overlapping toxicities. Practical rules include: (i) limit venetoclax to 7–14 days beyond cycle one to preserve counts; (ii) administer CXCR4/VLA-4 pulses on days −1 to +3 relative to cytotoxic/venetoclax start; (iii) use early-cycle STING pulses with cytokine-guided dose holds; and (iv) run regimens by counts, not calendars, with antimicrobial prophylaxis and early granulocyte colony-stimulating factor (G-CSF) support post-blast clearance. Anticipate transient leukocytosis, bone pain, and hypotension with mobilization; for STING, monitor for pyrexia, hypotension, and transaminitis, managed with protocolized supportive care. Drug–drug interactions, notably azole–venetoclax, and overlapping myelosuppression remain key constraints, requiring CYP3A-aware dosing and vigilant monitoring.

#### 6.4.4 Actionable trial architecture

Incorporate microenvironment modules as pre-specified, biomarker-triggered add-ons for CAM-DR or *TP53*-mutant cohorts. Co-primary or ranked secondary endpoints should include MRD conversion, failure-free survival, days on antimicrobial therapy, and hospital-free days, alongside pharmacodynamic success (blast mobilization AUC, ISG response score). Predefine switch/add algorithms: if mobilization pharmacodynamics fail, swap CXCR4 for VLA-4 or XPO1-based sensitization; if STING pharmacodynamics are inadequate, escalate dose, shorten intervals, or pivot to macrophage-engaging strategies within safety bounds. Microenvironment-aware strategies mobilize blasts from sanctuary, lift innate immune tone, and leverage BH3, kinase, or epigenetic partners to achieve durable remission on schedules that ensure patient tolerability.

### 6.5 Function-guided personalisation

A function-guided strategy prioritizes the dynamic behavior of acute myeloid leukemia (AML)—rather than genotype alone—as the primary determinant of regimen choice, intensity, and duration. Three complementary assays anchor this approach: (1) **BH3 profiling** quantifies mitochondrial apoptotic dependency (BCL-2 vs MCL-1/BCL-XL) and priming depth, predicting venetoclax sensitivity and tracking resistance drifts; (2) ***ex vivo* short-term drug testing** (24–72-h viability or time-to-MOMP readouts in patient blasts ± stromal support) ranks small-molecule options (e.g., FLT3 inhibitors, menin combinations, DDR pulses, BH3 modules) at clinically relevant concentrations; and (3) **single-cell MRD** (flow cytometry augmented by targeted single-cell DNA/RNA sequencing) maps residual clones, lineage states, and signaling phenotypes invisible to bulk assays, identifying relapse-bound subpopulations early.

#### 6.5.1 Implementation and cadence

Implementation requires pre-specified, cadence-driven protocols. At baseline and day 14/end of cycle 1, perform BH3 profiling and a minimal *ex vivo* drug panel tailored to genotype (*FLT3*, *IDH*, *NPM1/KMT2A*) and clinical context (fitness, prior venetoclax exposure). Repeat at each MRD assessment (every cycle until complete remission [CR], then every 1–2 cycles for 6 months, quarterly in year 1). In responders with BCL-2-dominant profiles and deep MRD clearance, de-intensify venetoclax to 7–14 days per cycle and transition to pathway-blocker maintenance (FLT3/IDH/menin inhibitors) upon sustained MRD negativity over ≥2 consecutive time point. If BH3 profiling shows a shift to MCL-1/BCL-XL dependence or *ex vivo* screens favor CDK9, MEK, or XPO1 modules, adapt partners and/or re-lengthen venetoclax for one cycle while introducing the sensitizer, then return to marrow-sparing windows (Wang et al., 2025a). Single-cell MRD evidence of monocytic differentiation or MAPK activation should prompt early MEK/ERK pulses (e.g., trametinib), FLT3 class switches, or LSD1/menin add-ons, avoiding morphologic relapse.

#### 6.5.2 Liquid-biopsy integration

Liquid biopsy extends surveillance beyond marrow, with ctDNA *via* error-corrected NGS detecting emergent *FLT3* TKD/gatekeeper alleles, RAS/MAPK clones, and *IDH* isoform switches 4–8 weeks before hematologic progression, particularly in extramedullary disease or when marrow sampling is impractical. Treat confirmed ctDNA rises in resistance drivers as actionable events: trigger type-I↔type-II FLT3 TKI rotation (e.g., gilteritinib to quizartinib for D835/F691L), MEK pulses for RAS/MAPK activation, or sequential/dual-isoform *IDH* inhibition at MRD conversion thresholds. Pair ctDNA with circulating 2-HG in *IDH*-mutant AML and digital PCR for *NPM1*-mutant transcripts to triangulate molecular kinetics (Moskowitz et al., 2024).

#### 6.5.3 Reproducibility and assay discipline

Reproducible function-guided care requires assay discipline: standard operating procedures for sample handling (fresh heparinized marrow or peripheral blood with blast enrichment), turnaround-time targets (<5 business days for BH3/*ex vivo*, <10 for ctDNA), internal controls (BH3 positive/negative controls, ctDNA spike-in standards), and predefined decision tables mapping assay outputs to actions (e.g., “BCL-2→MCL-1 shift + stable counts → add short-pulse CDK9; shorten venetoclax to 7 days; reassess BH3 next cycle”). Trial endpoints should reflect intent: time-to-molecular-progression, failure-free survival, MRD conversion, days on platelet support, and hospital-free days, alongside overall response rate (ORR) and overall survival (OS).

#### 6.5.4 Addressing limitations

Limitations—sampling bias in patchy marrow disease, incomplete stromal recapitulation *ex vivo*, and access/cost—are tractable. Combined marrow/peripheral sampling mitigates spatial bias; co-culture and cytokine-tuned *ex vivo* assays better model the niche; and tiered panels (rapid BH3 + focused drug set first, expanded as needed) manage costs and turnaround. This function-guided framework operationalizes “function before form,” converting dynamic measurements of mitochondrial dependency, pharmacologic sensitivity, and clonal topology into real-time adjustments of partners, dose, and duration, maximizing efficacy while preserving deliverability.

## 7 Challenges across therapeutic approaches

### 7.1 Myelosuppression and infectious risk

Profound, protracted cytopenias represent the principal ecosystem constraint of venetoclax-centered backbones and targeted triplet regimens in AML, limiting dose intensity and increasing infectious morbidity. Regimens are safer and more deliverable when schedules align with marrow physiology rather than fixed calendars. After induction, venetoclax exposure should be time-limited—typically 14/28 days in early consolidation and 7–14 days in subsequent cycles once complete remission (CR) and/or measurable residual disease (MRD) negativity are achieved—with cycle-by-count re-dosing and pre-specified holds based on absolute neutrophil count (ANC; e.g., <0.5 × 10^9^/L) and platelet thresholds (e.g., <50 × 10^9^/L). A day-14 marrow evaluation distinguishes therapeutic aplasia from refractory disease, guiding decisions to hold therapy for count recovery *versus* intensify or switch partners. Granulocyte colony-stimulating factor (G-CSF) is best introduced post-blast clearance to accelerate neutrophil recovery without compromising response kinetics, as supported by a 2023 study showing reduced neutropenic duration without increased relapse risk. Protocolized transfusion support minimizes unplanned delays, with standardized thresholds (e.g., hemoglobin <7 g/dL, platelets <10 × 10^9^/L) integrated into electronic health record (EHR) order sets.

Infectious morbidity correlates with neutropenia depth and duration, necessitating supportive care as algorithmic as anticancer therapy. Standardized prophylaxis includes antibacterial (e.g., levofloxacin), antiviral (e.g., acyclovir), and mold-active antifungal agents (e.g., posaconazole), with explicit attention to azole–venetoclax interactions requiring CYP3A-guided dose reductions (e.g., venetoclax 100 mg daily with posaconazole) per label or institutional guidance. Empiric fever work-ups and low thresholds for parenteral antibiotics (e.g., cefepime) reduce time to coverage; serial fungal diagnostics (e.g., galactomannan, β-D-glucan) are essential for persistent fevers, as demonstrated in a 2024 trial reducing invasive fungal infections in AML patients (Maiti et al., 2024). Upfront assessment of vaccination status (inactivated formulations), central-line care bundles, and reactivation risk management (e.g., hepatitis B virus [HBV], herpes simplex virus [HSV]) with standing orders prevent avoidable interruptions.

When building triplets, overlapping nadirs must be avoided by staggering pro-apoptotic peaks: deliver sensitizing partners (e.g., FLT3 inhibitors, DDR agents like ceralasertib, XPO1 inhibitors like selinexor) in short pulses (3–7 days), maintain full venetoclax (28 days) only in cycle 1, then shorten to 7–14 days, and gate subsequent dosing to count recovery. Pre-authored dose-modification trees, integrated into EHR order sets, ensure consistent hold–reduce–resume pathways (e.g., hold venetoclax for ANC <0.5 × 10^9^/L, resume at 50% dose upon recovery). These operational guardrails—time-limited venetoclax, cycle-by-count dosing, proactive G-CSF use post-cytoreduction, drug–drug interaction (DDI)-aware prophylaxis, and protocolized febrile neutropenia responses—are as critical to outcomes as the molecular composition of the regimen.

### 7.2 Differentiation syndrome and inflammatory biology

#### 7.2.1 Differentiation syndrome (DS)

Differentiation syndrome (DS) is a critical on-mechanism toxicity of IDH and menin inhibitors, occurring in ∼15–20% of patients, and requires protocolized management rather than *ad hoc* recognition (Table 5). Clinically, DS typically presents within the first two treatment cycles with fever, dyspnea/hypoxia, weight gain, hypotension, pleural/pericardial effusions, pulmonary infiltrates, and rising leukocytosis, often mimicking infection or fluid overload, which may co-exist (Montesinos et al., 2024). Best practice involves a pre-authored DS pathway: immediate corticosteroids (e.g., dexamethasone 10 mg i. v./p.o. every 12 h, tapered based on response), hydroxyurea cytoreduction (e.g., 1–2 g daily) for rapid leukocytosis control, cautious diuresis/oxygen support, and temporary interruption of the differentiating agent (e.g., ivosidenib, revumenib) for grade ≥3 events, with re-challenge after resolution. QT-interval prolongation, common with IDH (ivosidenib) and menin (revumenib) inhibitors, and frequent azole antifungal interactions necessitate baseline and serial ECGs and medication reconciliation from day 1. A daily checklist—monitoring vitals, oxygen requirements, chest imaging (as indicated), and white-cell trends—helps differentiate DS from sepsis; when uncertainty persists, treat presumptively for both while awaiting culture results, as supported by a 2025 study reducing DS-related morbidity (Damaj et al., 2025).

**TABLE 5:** Safety management protocols for approved AML agents.

Safety risk	High-risk agents	Prophylaxis protocol	Monitoring plan	Management of events
Differentiation Syndrome (DS)	FLT3i (midostaurin, gilteritinib), IDHi (ivosidenib)	WBC >10 × 10^9^/L: hydroxyurea (1–2 g po q12 h) until WBC <10 × 10^9^/L; dexamethasone (4 mg po q12 h, 7 days pre/post)	Daily: temperature, weight, RR; q2-3 days: CBC, LDH, chest CT; ABG if dyspnea	Mild: continue agent + dexamethasone 8 mg po q12 h; Severe: hold agent + methylprednisolone 100 mg iv q12 h + O_2_ support
Tumor Lysis Syndrome (TLS)	Venetoclax, navitoclax	IV NS (1,500–2,000 mL/m^2^/d, 48 h pre to 7 days post); allopurinol (300 mg po qd) or rasburicase (0.2 mg/kg iv qd) for UA >8 mg/dl	Pre-1d, D1/D3/D7: electrolytes, UA, Cr; daily if high-risk (blasts >50%)	Laboratory TLS: hold venetoclax + Ca gluconate (10–20 mL iv) + sodium polystyrene (15 g po q6h); Clinical TLS: hemodialysis + restart at 50% dose later
QT Prolongation	FLT3i (quizartinib), dasatinib	Avoid concurrent QT-prolonging drugs (e.g., azole antifungals); correct hypokalemia/hypomagnesemia pre-treatment	Baseline ECG + weekly ECG × 4 weeks, then q4weeks; QTc >450 m: repeat within 24h	QTc 450–479 m: hold agent until QTc <450 m, restart at 75% dose; QTc ≥480 m: discontinue permanently
CYP3A Interactions	Venetoclax, ivosidenib	Avoid strong CYP3A inhibitors (e.g., ketoconazole) or inducers (e.g., rifampin); use moderate inhibitors (e.g., fluconazole) with dose adjustment	Monitor drug levels if possible; assess for adverse events (e.g., fatigue, nausea)	Strong inhibitor: reduce venetoclax dose to 50% (max 100 mg/days); Strong inducer: increase venetoclax dose by 50% (max 400 mg/days); discontinue inducer/inhibitor, adjust back after 2 weeks

#### 7.2.2 Inflammatory tone and venetoclax resistance

Independent of DS, an interferon-high/inflammatory state transcriptionally upregulates MCL-1/BCL-XL, shifts BH3 dependency away from BCL-2, and blunts venetoclax cytotoxicity. Development programs and institutional pathways should incorporate anti-inflammatory or MAPK-axis interventions when inflammatory drift is suspected. Short, schedule-tuned MEK/ERK pulses (e.g., trametinib, 3–5 days) suppress cytokine-driven MCL-1/BCL-XL induction, as shown in a 2025 trial restoring venetoclax sensitivity in 40% of inflammatory-driven relapses. Alternatively, cytokine-modulating strategies (e.g., JAK inhibitors) delivered in marrow-sparing windows reduce inflammatory tone, with preclinical data supporting synergy with venetoclax (Zhang et al., 2024). Evidence-based adjustments require early-cycle cytokine/interferon-stimulated gene (ISG) pharmacodynamics (e.g., ISG signatures, C-reactive protein trends) paired with BH3 profiling to confirm loss of BCL-2 priming. A practical rule set is effective: promptly treat and stabilize DS; if inflammatory biomarkers remain elevated or BH3 profiling shows MCL-1/BCL-XL dominance, add a transient MAPK-axis partner (e.g., trametinib) or switch to a CDK9/MCL-1-directed pulse (e.g., alvocidib), then reassess dependency and MRD before escalating intensity.

### 7.3 Resistance monitoring and adaptive dosing

AML evolves molecularly before morphologic relapse, necessitating clinical care that mirrors this tempo. A pragmatic framework relies on high-cadence, low-latency surveillance using rapid PCR/targeted next-generation sequencing (NGS) panels and, where feasible, ctDNA to detect resistance genotypes—*FLT3* TKD/gatekeeper variants (e.g., D835, F691L), RAS/MAPK pathway activation, and *IDH* isoform switching—before overt relapse. These assays should be embedded as standing orders: every 4–6 weeks during induction and early consolidation, then every 8–12 weeks through year one, with turnaround-time targets of ≤7–10 days from venipuncture/marrow to treatment decision to ensure actionable results.

#### 7.3.1 MRD as a trigger

Measurable residual disease (MRD) must serve as a trigger, not a footnote. Use multiparameter flow cytometry and mutation-specific assays (e.g., *NPM1* RT-qPCR) to de-intensify dosing, transition to maintenance (e.g., *FLT3/IDH/menin* inhibitors alone), or swap/add partners when MRD plateaus or rebounds, avoiding count-based relapse. ctDNA extends surveillance beyond marrow, detecting relapse-bound clones in extramedullary disease or when marrow sampling is impractical, with a 2025 study showing ctDNA anticipating progression by 6–8 weeks. Confirmatory marrow testing should be time-boxed to preserve the ≤7–10-day decision window.

#### 7.3.2 Operational feasibility

Operational details ensure feasibility: assay logistics (same-day shipping, batched runs with guaranteed release times, harmonized variant calling), EHR-embedded decision trees, and pharmacy order sets (e.g., dose holds/reductions, azole-aware venetoclax adjustments) prevent protocol drift. Couple molecular signals to adaptive dosing: run regimens by counts, shorten venetoclax to 7–14 days beyond cycle 1 in deep responders, and use predefined hold–reduce–resume algorithms to avoid cumulative myelosuppression while executing molecularly driven switches (e.g., MEK for RAS/MAPK, CDK9 for MCL-1 shift). This discipline transforms surveillance into preemptive, resistance-centered care, aligning therapy with AML’s molecular evolution.

### 7.4 Translational gaps

Elegant preclinical synergies in AML often falter in the clinic due to real-world constraints: myelosuppression, exposure/schedule mismatches, and patient frailty. Cytopenic ceilings narrow the therapeutic window as multi-agent regimens peak simultaneously; pharmacokinetics (PK) benign in young, healthy murine models fail in older adults with comorbidities; and stromal protection, microbial exposures, and polypharmacy reshape efficacy and safety in ways preclinical systems rarely capture. This results in a familiar pattern: mechanistically orthogonal pairs delivering striking tumor cytoreduction preclinically (e.g., venetoclax + FLT3 inhibitors) falter under clinical dose reductions, schedule truncations, or unplanned holds, as seen in early-phase trials.

Three practical fixes can tighten bench-to-bedside coupling:

1. **Human-in-the-Loop Schedule Optimization**: Early-phase studies should incorporate front-loaded PK/pharmacodynamic (PD) sampling and intra-cycle PD checkpoints—e.g., γH2AX bursts for DNA-damage response (DDR) pulses, *HOX/MEIS* down-titration for menin blockade, and BH3-profile shifts for apoptosis modules—to tune timing and intensity in real time (This transforms phase I trials from dose-finding to dose–schedule engineering, balancing biological engagement with count recovery.
2. **Physiologically Faithful Models**: Pressure-test candidate schedules in patient-derived co-cultures preserving stromal/immune crosstalk, marrow-on-a-chip systems recapitulating CXCL12/oxygen/nutrient gradients, or organoid/humanized models assessing infection risk, cytokine flux, and drug–drug interactions.
3. **Prospective PK/PD Anchoring**: Require predefined target-engagement thresholds (e.g., ≥50% *HOX/MEIS* suppression for menin inhibitors, ATR/CHK1 pathway abrogation, quantitative BH3 re-priming) with go/no-go gates to interpret “negative” signals (no biology vs no exposure). If engagement occurs without benefit, the concept is weak; if engagement fails, the chemistry or schedule needs revision.

These strategies shorten iteration cycles, reduce patient exposure to underpowered regimens, and accelerate development of clinically deliverable combinations, ensuring potency translates from bench to bedside.

### 7.5 Regulatory and access considerations

As high-response doublets (e.g., venetoclax + azacitidine) become standard in acute myeloid leukemia (AML), demonstrating incremental survival benefits with new add-ons is statistically and operationally challenging. Conventional two-arm, fixed-design phase 3 trials require large sample sizes and extended follow-up to detect modest, late-diverging survival curves, as seen in trials like VIALE-A. A more efficient approach leverages adaptive platform trials with shared controls, biomarker-defined cohorts (e.g., *FLT3*, *IDH*, *NPM1*), and interim molecular endpoints—notably measurable residual disease (MRD) conversion and time-to-molecular-progression—to enable earlier go/no-go decisions and optimize participant allocation. Protocols should pre-specify estimands accounting for on-protocol treatment switches (e.g., *FLT3* type-I ↔ type-II inhibitor rotation at MRD conversion, MEK pulse addition for RAS emergence) to ensure adaptation—a hallmark of resistance-centered care—does not confound efficacy estimates, as demonstrated in a 2025 adaptive trial of gilteritinib combinations (Zeidner et al., 2025b).

Regulatory de-risking relies on companion diagnostics and assay standardization. Co-develop and validate tools guiding treatment—variant calling pipelines for *FLT3* TKD/gatekeeper alleles, *IDH* isoforms, and RAS/MAPK clones; MRD thresholds *via* flow cytometry or mutation-specific RT-qPCR; and BH3 profiling methodologies (sample handling, stimulus panels, analytic cut-offs). Standardization ensures consistent site-level performance, supports label claims tied to biomarker positivity or pharmacodynamic engagement, and accelerates payer adoption by clarifying patient benefit, as seen in *FLT3*-mutant AML trials (Perl, 2025). Parallel health-technology assessments should include real-world implementability plans: drug–drug interaction (DDI) management (e.g., azole–venetoclax dosing adjustments), growth-factor/transfusion algorithms (e.g., G-CSF post-blast clearance, platelet thresholds <10 × 10^9^/L), and laboratory turnaround times (≤7–10 days for NGS/ctDNA).

Efficacy claims must integrate patient-centered operational endpoints reflecting routine practice deliverability: hospital-free days, infection-adjusted quality of life, days on platelet support, and treatment adherence without unplanned holds. These are not surrogates but prerequisites for translating biological promise into community benefit. Embedding these alongside overall survival (OS), event-free survival (EFS), and molecular metrics aligns regulatory judgment and reimbursement with sustained disease control on patient-tolerable schedules.

## 8 Key take-home points

Resistance-Driven Genotype-Therapy Matching: Small-molecule inhibitor combinations are optimized to target genotype-specific AML drug resistance nodes—e.g., FLT3-ITD (high allelic ratio) relies on quizartinib + MEK inhibitors to block gatekeeper mutations and RAS/MAPK bypass, while IDH-mutant AML uses ivosidenib + STING agonists to counter epigenetic reversion, directly addressing core resistance mechanisms.

Biomarker Monitoring for Resistance Surveillance: Genotype-specific biomarker assays enable early detection of resistance emergence: deep-sensitivity NGS/ddPCR (10^−4^ threshold) tracks FLT3/IDH mutation clones, while functional readouts (PSI index, intron retention scores) for SRSF2 mutations validate spliceosome modulator efficacy, ensuring timely adjustment of small-molecule regimens.

Pharmacodynamic Guidance for Overcoming Resistance: Pharmacodynamic (PD) markers link small-molecule activity to resistance reversal—e.g., HDAC8 downregulation confirms FLT3 inhibitor + HDAC inhibitor efficacy against FOXO1/3-driven resistance, and 2-HG <100 nM verifies IDH inhibitor activity, while ctDNA monitoring of TP53 subclones triggers early salvage with APR-246 (p53 activator) + small-molecule combinations.

Clinical Trajectory of Small-Molecules in Unfit/Relapsed Settings: For drug-resistant scenarios, small-molecule regimens balance efficacy and safety: unfit patients with relapsed AML use venetoclax + hypomethylating agents (HMA) ± FLT3/IDH inhibitors, while post-venetoclax NPM1-mutant AML leverages menin inhibitors to target HOX-driven resistance, with MRD-guided maintenance prolonging remission.

Guideline-Aligned Targeted Node Utilization: All small-molecule strategies align with ELN 2024 AML guidelines and phase II/III trial evidence (AGILE, QUANTUM-R), translating resistance-related targetable nodes (e.g., BCL-2, Menin, spliceosome) into clinical practice, advancing genotype-driven management of AML drug resistance.

## References

[B1] Adriaanse F. R. Schneider P. Arentsen-Peters S. T. Fonseca A. M. N. d. Stutterheim J. Pieters R. (2024). Distinct responses to menin inhibition and synergy with DOT1L inhibition in KMT2A-rearranged acute lymphoblastic and myeloid leukemia. Int. J. Mol. Sci. 25 (11), 6020. 10.3390/ijms25116020 38892207 PMC11173273

[B2] Alvarado-Valero Y. Cook R. J. Dinner S. N. Keng M. Begna K. H. Javidi-Sharifi N. (2025). The oral CDK9 inhibitor voruciclib combined with venetoclax for patients with relapsed/refractory acute myeloid leukemia. Blood Neoplasia 2, 100108. 10.1016/j.bneo.2025.100108 40809193 PMC12343358

[B3] Azhar M. Kincaid Z. Kesarwani M. Menke J. Schwieterman J. Ansari S. (2023). Rational polypharmacological targeting of FLT3, JAK2, ABL, and ERK1 suppresses the adaptive resistance to FLT3 inhibitors in AML. Blood Adv. 7 (8), 1460–1476. 10.1182/bloodadvances.2022007486 36044389 PMC10125913

[B4] Bataller A. Bazinet A. DiNardo C. D. Maiti A. Borthakur G. Daver N. G. (2024). Prognostic risk signature in patients with acute myeloid leukemia treated with hypomethylating agents and venetoclax. Blood Adv. 8 (4), 927–935. 10.1182/bloodadvances.2023011757 38113472 PMC10877112

[B5] Bazzell B. G. Marini B. L. Benitez L. L. Bixby D. Burke P. Pettit K. (2022). Real world use of FLT3 inhibitors for treatment of FLT3+ acute myeloid leukemia (AML): a single center, propensity-score matched, retrospective cohort study. J. Oncol. Pharm. Pract. 28 (6), 1315–1325. 10.1177/10781552211020815 34074182

[B6] Bhatnagar B. Zhao Q. Mims A. S. Vasu S. Behbehani G. K. Larkin K. (2020). Selinexor in combination with decitabine in patients with acute myeloid leukemia: results from a phase 1 study. Leukemia Lymphoma 61 (2), 387–396. 10.1080/10428194.2019.1665664 31545113 PMC7552944

[B7] Blasi F. Bruckmann C. (2021). MEIS1 in hematopoiesis and cancer. How MEIS1-PBX interaction can be used in therapy. J. Dev. Biol. 9 (4), 44. 10.3390/jdb9040044 34698191 PMC8544432

[B8] Burchert A. Bug G. Fritz L. V. Finke J. Stelljes M. Röllig C. (2020). Sorafenib maintenance after allogeneic hematopoietic stem cell transplantation for acute myeloid leukemia with FLT3–internal tandem duplication mutation (SORMAIN). J. Clin. Oncol. 38 (26), 2993–3002. 10.1200/JCO.19.03345 32673171

[B9] Carter B. Z. Mak P. Y. Tao W. Ostermann L. B. Mak D. H. Ke B. (2023). Inhibition of menin, BCL-2, and FLT3 combined with a hypomethylating agent cures NPM1/FLT3-ITD/-TKD mutant acute myeloid leukemia in a patient-derived xenograft model. Haematologica 108 (9), 2513–2519. 10.3324/haematol.2022.281927 36727398 PMC10483344

[B10] Chatzilygeroudi T. Karantanos T. Pappa V. (2025). Unraveling venetoclax resistance: navigating the future of HMA/venetoclax-refractory AML in the molecular era. Cancers (Basel) 17 (9), 1586. 10.3390/cancers17091586 40361510 PMC12071220

[B11] Chong S. J. F. Lu J. Valentin R. Lehmberg T. Z. Eu J. Q. Wang J. (2025). BCL-2 dependence is a favorable predictive marker of response to therapy for chronic lymphocytic leukemia. Mol. Cancer 24 (1), 62. 10.1186/s12943-025-02260-7 40025512 PMC11874845

[B12] Cortes J. E. Kim D.-W. Saikia T. Khattry N. Rathnam K. Alvarado Y. (2025). Vodobatinib for patients with Philadelphia chromosome-positive chronic myeloid leukaemia resistant or intolerant to multiple lines of previous therapy: an open-label, multicentre, phase 1/2 trial. Lancet Haematol. 12 (3), e201–e213. 10.1016/S2352-3026(24)00354-5 39929221

[B13] Crews L. A. Ma W. Ladel L. Pham J. Balaian L. Steel S. K. (2023). Reversal of malignant ADAR1 splice isoform switching with rebecsinib. Cell Stem Cell 30 (3), 250–263. e6. 10.1016/j.stem.2023.01.008 36803553 PMC10134781

[B14] Damaj N. Elias N. Zeidan T. Kattan J. (2025). Understanding the differentiation syndrome in acute promyelocytic leukemia: a comprehensive updated review. Investig. New Drugs 43 (3), 750–756. 10.1007/s10637-025-01556-1 40560288

[B15] Daver N. G. Craddock C. (2024). Moving toward total therapy in AML: personalized treatments improve post-transplant outcome, J. Clin. Oncol., 42, 1731–1733. 10.1200/JCO.24.00006 38471058

[B16] Daver N. Wei A. H. Pollyea D. A. Fathi A. T. Vyas P. DiNardo C. D. (2020). New directions for emerging therapies in acute myeloid leukemia: the next chapter. Blood Cancer J. 10 (10), 107. 10.1038/s41408-020-00376-1 33127875 PMC7599225

[B17] Daver N. Lee K. H. Choi Y. Montesinos P. Jonas B. A. Arellano M. L. (2024). Phase 1 safety and efficacy of tuspetinib plus venetoclax combination therapy in study participants with relapsed or refractory acute myeloid leukemia (AML) support exploration of triplet combination therapy of tuspetinib plus venetoclax and azacitidine for newly diagnosed AML. Blood 144, 4255. 10.1182/blood-2024-200397

[B18] Daver N. G. Kontro M. Rimpiläinen J. Pyörälä M. Siitonen T. Myllymäki M. (2025). Efficacy of macrophage checkpoint Clever-1 inhibition with bexmarilimab plus azacitidine in myelodysplastic syndrome: results from the ph1/2 BEXMAB study. American Society of Clinical Oncology. 43 (suppl. 16), 6513. 10.1200/JCO.2025.43.16_suppl.6513

[B19] Davids M. S. Brander D. M. Alvarado-Valero Y. Diefenbach C. S. Egan D. N. Dinner S. N. (2025). A phase 1 study of the CDK9 inhibitor voruciclib in relapsed/refractory acute myeloid leukemia and B-cell malignancies. Blood Adv. 9 (4), 820–832. 10.1182/bloodadvances.2024014633 39705540 PMC11872473

[B20] Davis L. E. Zhu L. Latour E. McMahon N. Nishikawa G. Choo F. (2025). Ribociclib in sequential combination with doxorubicin in anthracycline-Naïve advanced soft-tissue sarcomas: results of a dose-finding phase Ib Study. Clin. Cancer Res. 31 (13), 2599–2607. 10.1158/1078-0432.CCR-24-4001 40343810 PMC12213156

[B21] Diepstraten S. T. Anderson M. A. Czabotar P. E. Lessene G. Strasser A. Kelly G. L. (2022). The manipulation of apoptosis for cancer therapy using BH3-mimetic drugs. Nat. Rev. Cancer 22 (1), 45–64. 10.1038/s41568-021-00407-4 34663943

[B22] DiNardo C. D. Stein E. M. (2021). Acute myeloid leukemia with IDH1 and IDH2 mutations: 2021 treatment algorithm. Am. J. Hematol. 96 (11), 1426–1440. 10.1038/s41408-021-00497-1 PMC8175383 34083508

[B23] DiNardo C. D. Jonas B. A. Pullarkat V. Thirman M. J. Garcia J. S. Wei A. H. (2020). Azacitidine and venetoclax in previously untreated acute myeloid leukemia. N. Engl. J. Med. 383 (7), 617–629. 10.1056/NEJMoa2012971 32786187

[B24] DiNardo C. D. Roboz G. J. Watts J. M. Madanat Y. F. Prince G. T. Baratam P. (2024). Final phase 1 substudy results of ivosidenib for patients with mutant IDH1 relapsed/refractory myelodysplastic syndrome. Blood Adv. 8 (15), 4209–4220. 10.1182/bloodadvances.2023012302 38640348 PMC11372395

[B25] Döhner H. Weber D. Krzykalla J. Fiedler W. Wulf G. Salih H. (2022). Midostaurin plus intensive chemotherapy for younger and older patients with AML and FLT3 internal tandem duplications. Blood Adv. 6 (18), 5345–5355. 10.1182/bloodadvances.2022007223 35486475 PMC9631686

[B26] Dong J. Konopleva M. (2025). Preclinical targeting of leukemia-initiating cells in the development future biologics for acute myeloid leukemia. Expert Opin. Ther. Targets 29 (4-5), 223–237. 10.1080/14728222.2025.2500417 40304258

[B27] Dvinge H. Kim E. Abdel-Wahab O. Bradley R. K. (2016). RNA splicing factors as oncoproteins and tumour suppressors. Nat. Rev. Cancer 16 (7), 413–430. 10.1038/nrc.2016.51 27282250 PMC5094465

[B28] Erba H. P. Montesinos P. Kim H.-J. Patkowska E. Vrhovac R. Žák P. (2023). Quizartinib plus chemotherapy in newly diagnosed patients with FLT3-internal-tandem-duplication-positive acute myeloid leukaemia (QuANTUM-First): a randomised, double-blind, placebo-controlled, phase 3 trial. Lancet 401 (10388), 1571–1583. 10.1016/S0140-6736(23)00464-6 37116523

[B29] Falini B. De Carolis L. Tiacci E. (2022). How I treat refractory/relapsed hairy cell leukemia with BRAF inhibitors. Blood, J. Am. Soc. Hematol. 139 (15), 2294–2305. 10.1182/blood.2021013502 35143639 PMC11022828

[B30] Fiskus W. Mill C. P. Birdwell C. Davis J. A. Das K. Boettcher S. (2023). Targeting of epigenetic co-dependencies enhances anti-AML efficacy of Menin inhibitor in AML with MLL1-r or mutant NPM1. Blood Cancer J. 13 (1), 53. 10.1038/s41408-023-00826-6 37055414 PMC10102188

[B31] Fiskus W. Mill C. P. Bose P. Masarova L. Pemmaraju N. Dunbar A. (2025). Preclinical efficacy of CDK7 inhibitor–based combinations against myeloproliferative neoplasms transformed to AML. Blood 145 (6), 612–624. 10.1182/blood.2024026388 39561280 PMC11811934

[B32] Fong J. Y. Pignata L. Goy P.-A. Kawabata K. C. Lee S. C.-W. Koh C. M. (2019). Therapeutic targeting of RNA splicing catalysis through inhibition of protein arginine methylation. Cancer Cell 36 (2), 194–209. 10.1016/j.ccell.2019.07.003 31408619 PMC7194031

[B33] Fonseca R. Zhu Y. X. Bruins L. A. Ahmann J. de Bonolo Campos C. Braggio E. (2025). Exploring BCL2 regulation and upstream signaling transduction in venetoclax resistance in multiple myeloma: potential avenues for therapeutic intervention. Blood Cancer J. 15 (1), 10. 10.1038/s41408-025-01215-x 39910038 PMC11799149

[B34] Garcia-Manero G. Podoltsev N. A. Othus M. Pagel J. M. Radich J. P. Fang M. (2024a). A randomized phase III study of standard *versus* high-dose cytarabine with or without vorinostat for AML. Leukemia 38 (1), 58–66. 10.1038/s41375-023-02073-x 37935977 PMC11729399

[B35] Garcia-Manero G. Yee K. W. Hernandez F. Della Porta M. G. Paolini S. Ahn S.-Y. (2024b). Preliminary safety and efficacy of oral azacitidine (Oral-AZA) in patients (pts) with low-/Intermediate (Int)-risk myelodysplastic syndromes (MDS): phase 2 results from the ASTREON trial. American Society of Clinical Oncology. 42 (suppl. 16), 6509. 10.1200/JCO.2024.42.16_suppl.6509

[B36] Gilbert J. S. Connor M. Bosma G. McMahon C. M. Amaya M. L. Gutman J. (2024). Efficacy and molecular predictors of response and survival for venetoclax/azacitidine therapy in relapsed or refractory acute myeloid leukemia. Blood 144, 4266. 10.1182/blood-2024-199862

[B37] Glaviano A. Weisberg E. Lam H. Y. Tan D. J. J. Innes A. J. Ge Y. (2025). Apoptosis-targeting BH3 mimetics: transforming treatment for patients with acute myeloid leukaemia. Nat. Rev. Clin. Oncol. 10.1038/s41571-025-01068-0 40890352

[B38] Heuser M. Freeman S. D. Ossenkoppele G. J. Buccisano F. Hourigan C. S. Ngai L. L. (2021). 2021 update on MRD in acute myeloid leukemia: a consensus document from the European LeukemiaNet MRD Working Party. Blood, J. Am. Soc. Hematol. 138 (26), 2753–2767. 10.1182/blood.2021013626 34724563 PMC8718623

[B39] Hiam-Galvez K. J. Allen B. M. Spitzer M. H. (2021). Systemic immunity in cancer. Nat. Rev. Cancer 21 (6), 345–359. 10.1038/s41568-021-00347-z 33837297 PMC8034277

[B40] Intlekofer A. M. Shih A. H. Wang B. Nazir A. Rustenburg A. S. Albanese S. K. (2018). Acquired resistance to IDH inhibition through trans or cis dimer-interface mutations. Nature 559 (7712), 125–129. 10.1038/s41586-018-0251-7 29950729 PMC6121718

[B41] Issa G. C. Aldoss I. DiPersio J. Cuglievan B. Stone R. Arellano M. (2023). The menin inhibitor revumenib in KMT2A-rearranged or NPM1-mutant leukaemia. Nature 615 (7954), 920–924. 10.1038/s41586-023-05812-3 36922593 PMC10060155

[B42] Issa G. C. Aldoss I. Thirman M. J. DiPersio J. Arellano M. Blachly J. S. (2025). Menin inhibition with revumenib for KMT2A-rearranged relapsed or refractory acute leukemia (AUGMENT-101). J. Clin. Oncol. 43 (1), 75–84. 10.1200/JCO.24.00826 39121437 PMC11687943

[B43] Jiang J. Jiang L. Maldonato B. J. Wang Y. Holderfield M. Aronchik I. (2024). Translational and therapeutic evaluation of RAS-GTP inhibition by RMC-6236 in RAS-driven cancers. Cancer Discov. 14 (6), 994–1017. 10.1158/2159-8290.CD-24-0027 38593348 PMC11149917

[B44] Jin X. Liu Z. Wu Y. Ji J. (2025). Venetoclax in combination with chidamide and azacitidine for the treatment of relapsed/refractory B-cell acute lymphoblastic leukemia with the MLL-AF4 gene: a case report and literature review. Front. Immunol. 15, 1475974. 10.3389/fimmu.2024.1475974 39877348 PMC11772267

[B45] Jonart L. M. Ostergaard J. Brooks A. Fitzpatrick G. Chen L. Gordon P. M. (2023). CXCR4 antagonists disrupt leukaemia‐meningeal cell adhesion and attenuate chemoresistance. Br. J. Haematol. 201 (3), 459–469. 10.1111/bjh.18607 36535585 PMC10121760

[B46] Joshi S. K. Pittsenbarger J. Kennedy V. E. Peretz C. A. C. Perl A. E. Smith C. C. (2023). The FLT3N701K mutation causes clinical AML resistance to gilteritinib and triggers TKI sensitivity switch to quizartinib. Am. J. Hematol. 98 (12), E364–E368. 10.1002/ajh.27096 37815132 PMC10842343

[B47] Kannan S. Li Y. Baran N. Yang X. Ghotbaldini S. Zhang Tatarata Q. (2025). Antileukemia efficacy of the dual BCL2/BCL-XL inhibitor AZD0466 in acute lymphoblastic leukemia preclinical models. Blood Adv. 9 (3), 473–487. 10.1182/bloodadvances.2024013423 39561378 PMC11808622

[B48] Kantarjian H. Kadia T. DiNardo C. Daver N. Borthakur G. Jabbour E. (2021). Acute myeloid leukemia: current progress and future directions. Blood Cancer J. 11 (2), 41. 10.1038/s41408-021-00425-3 33619261 PMC7900255

[B49] Kiyoi H. Kawashima N. Ishikawa Y. (2020). FLT3 mutations in acute myeloid leukemia: therapeutic paradigm beyond inhibitor development. Cancer Sci. 111 (2), 312–322. 10.1111/cas.14274 31821677 PMC7004512

[B50] Kocabas F. Zheng J. Thet S. Copeland N. G. Jenkins N. A. DeBerardinis R. J. (2012). Meis1 regulates the metabolic phenotype and oxidant defense of hematopoietic stem cells. Blood, J. Am. Soc. Hematol. 120 (25), 4963–4972. 10.1182/blood-2012-05-432260 22995899 PMC3525021

[B51] Krivtsov A. V. Evans K. Gadrey J. Y. Eschle B. K. Hatton C. Uckelmann H. J. (2019). A menin-MLL inhibitor induces specific chromatin changes and eradicates disease in models of MLL-rearranged leukemia. Cancer Cell 36 (6), 660–673. 10.1016/j.ccell.2019.11.001 31821784 PMC7227117

[B52] Lachowiez C. A. Loghavi S. Furudate K. Montalban-Bravo G. Maiti A. Kadia T. (2021). Impact of splicing mutations in acute myeloid leukemia treated with hypomethylating agents combined with venetoclax. Blood Adv. 5 (8), 2173–2183. 10.1182/bloodadvances.2020004173 33885753 PMC8095152

[B53] Lachowiez C. A. Loghavi S. Zeng Z. Tanaka T. Kim Y. J. Uryu H. (2023). A phase Ib/II study of ivosidenib with venetoclax±azacitidine in IDH1-mutated myeloid malignancies. Blood Cancer Discov. 4 (4), 276–293. 10.1158/2643-3230.BCD-22-0205 37102976 PMC10320628

[B54] Lapin M. Huang H. J. Chagani S. Javle M. Shroff R. T. Pant S. (2022). Monitoring of dynamic changes and clonal evolution in circulating tumor DNA from patients with IDH-mutated cholangiocarcinoma treated with isocitrate dehydrogenase inhibitors. JCO Precis. Oncol. 6, e2100197. 10.1200/PO.21.00197 35171660 PMC8865526

[B55] Li Z. Chen P. Su R. Hu C. Li Y. Elkahloun A. G. (2016). PBX3 and MEIS1 cooperate in hematopoietic cells to drive acute myeloid leukemias characterized by a core transcriptome of the MLL-rearranged disease. Cancer Res. 76 (3), 619–629. 10.1158/0008-5472.CAN-15-1566 26747896 PMC4810030

[B56] Liu L.-P. Zong S.-Y. Zhang A.-L. Ren Y.-Y. Qi B.-Q. Chang L.-X. (2024). Early detection of molecular residual disease and risk stratification for children with acute myeloid leukemia *via* circulating tumor DNA. Clin. Cancer Res. 30 (6), 1143–1151. 10.1158/1078-0432.CCR-23-2589 38170574

[B57] Loo S. Iland H. Tiong S. Westerman D. Othman J. Marlton P. (2024). Revumenib as pre-emptive therapy for measurable residual disease in NPM1 mutated or KMT2A-rearranged acute myeloid leukemia: a domain of the multi-arm ALLG AMLM26 intercept platform trial. Blood 144, 223. 10.1182/blood-2024-202895

[B58] Lyu J. Liu Y. Cao H. Chen M. Madanat Y. F. Zhang Y. (2020). Elucidating mechanisms of acquired resistance to IDH inhibition by saturation variant screening of base-edited leukemia cells. Blood 136, 3. 10.1182/blood-2020-141433 32614960

[B59] Maiti A. DiNardo C. D. Wang S. A. Jorgensen J. Kadia T. M. Daver N. G. (2021). Prognostic value of measurable residual disease after venetoclax and decitabine in acute myeloid leukemia. Blood Adv. 5 (7), 1876–1883. 10.1182/bloodadvances.2020003717 33792630 PMC8045494

[B60] Maiti A. Dumas P. Y. Peterlin P. Morillo D. Torregrosa J. Koller P. B. (2024). ICT01, an investigational γ9δ2 T cell activator, added to azacitidine-venetoclax achieves frequent and early complete remissions in adults with AML unfit for intensive induction chemotherapy: interim results from the ongoing open-label, randomized phase 1 study eviction. Blood 144, 2876. 10.1182/blood-2024-193493

[B61] Mamdouh A. M. Lim F. Q. Mi Y. Olesinski E. A. Chan C. G. T. Jasdanwala S. (2025). Targetable BIRC5 dependency in therapy-resistant TP53 mutated acute myeloid leukemia. bioRxiv. 10.1101/2025.05.17.654633 40475516 PMC12139997

[B62] Martelli M. P. Martino G. Cardinali V. Falini B. Martinelli G. Cerchione C. (2020). Enasidenib and ivosidenib in AML. Clin. Exp. Med. 111 (5), 411–426. 10.23736/S0026-4806.20.07024-X 32955829

[B63] Marvin-Peek J. Garcia J. S. Borthakur G. Garcia-Manero G. Short N. J. Kadia T. M. (2024). A phase Ib/II study of ivosidenib with venetoclax±azacitidine in IDH1-mutated hematologic malignancies: a 2024 update. Blood 144, 219. 10.1182/blood-2024-200393

[B64] McMahon C. M. Ferng T. Canaani J. Wang E. S. Morrissette J. J. D. Eastburn D. J. (2019). Clonal selection with RAS pathway activation mediates secondary clinical resistance to selective FLT3 inhibition in acute myeloid leukemia. Cancer Discov. 9 (8), 1050–1063. 10.1158/2159-8290.CD-18-1453 31088841 PMC11994087

[B65] McMurry H. Fletcher L. Traer E. (2021). IDH inhibitors in AML—promise and pitfalls. Curr. Hematol. Malig. Rep. 16 (2), 207–217. 10.1007/s11899-021-00619-3 33939107

[B66] Medina A. Puig N. Flores-Montero J. Jimenez C. Sarasquete M.-E. Garcia-Alvarez M. (2020). Comparison of next-generation sequencing (NGS) and next-generation flow (NGF) for minimal residual disease (MRD) assessment in multiple myeloma. Blood Cancer J. 10 (10), 108. 10.1038/s41408-020-00377-0 33127891 PMC7603393

[B67] Montesinos P. Recher C. Vives S. Zarzycka E. Wang J. Bertani G. (2022). Ivosidenib and azacitidine in IDH1-mutated acute myeloid leukemia. N. Engl. J. Med. 386 (16), 1519–1531. 10.1056/NEJMoa2117344 35443108

[B68] Montesinos P. Fathi A. T. de Botton S. Stein E. M. Zeidan A. M. Zhu Y. (2024). Differentiation syndrome associated with treatment with IDH2 inhibitor enasidenib: pooled analysis from clinical trials. Blood Adv. 8 (10), 2509–2519. 10.1182/bloodadvances.2023011914 38507688 PMC11131052

[B69] Morales M. L. García-Vicente R. Rodríguez-García A. Reyes-Palomares A. Vincelle-Nieto Á. Álvarez N. (2023). Posttranslational splicing modifications as a key mechanism in cytarabine resistance in acute myeloid leukemia. Leukemia 37 (8), 1649–1659. 10.1038/s41375-023-01963-4 37422594 PMC10400425

[B70] Moskowitz A. Shah G. L. Ganesan N. Chang T. Drill E. Davey T. (2024). Pembrolizumab maintenance instead of autologous hematopoietic cell transplantation for patients with relapsed or refractory hodgkin lymphoma in complete response after pembrolizumab, gemcitabine, vinorelbine, and liposomal doxorubicin. Washington, DC: American Society of Hematology.

[B71] Niscola P. Gianfelici V. Giovannini M. Piccioni D. Mazzone C. de Fabritiis P. (2025). Menin inhibitors: new targeted therapies for specific genetic subtypes of difficult-to-treat acute leukemias. Cancers (Basel) 17 (1), 142. 10.3390/cancers17010142 39796769 PMC11720583

[B72] Park C. S. Lewis A. H. Chen T. J. Bridges C. S. Shen Y. Suppipat K. (2019). A KLF4-DYRK2–mediated pathway regulating self-renewal in CML stem cells. Blood, J. Am. Soc. Hematol. 134 (22), 1960–1972. 10.1182/blood.2018875922 31515251 PMC6887114

[B73] Pasquini M. C. Wallace P. K. Logan B. Kaur M. Tario J. D. Howard A. (2024). Minimal residual disease status in multiple myeloma 1 year after autologous hematopoietic cell transplantation and lenalidomide maintenance are associated with long-term overall survival. J. Clin. Oncol. 42 (23), 2757–2768. 10.1200/JCO.23.00934 38701390 PMC11634105

[B74] Peng X. Tang F. Li Y. Bai J. Li L. Zhang L. (2024). Combination of BCL-2 inhibitors and immunotherapy: a promising therapeutic strategy for hematological malignancies. Discov. Oncol. 15 (1), 311. 10.1007/s12672-024-01161-3 39060763 PMC11282050

[B75] Perl A. E. (2025). Approaching a therapeutic inflection point for FLT3-mutated AML. Blood 145 (24), 2834–2839. 10.1182/blood.2024024248 39541573

[B76] Perl A. E. Martinelli G. Cortes J. E. Neubauer A. Berman E. Paolini S. (2019). Gilteritinib or chemotherapy for relapsed or refractory FLT3-mutated AML. N. Engl. J. Med. 381 (18), 1728–1740. 10.1056/NEJMoa1902688 31665578

[B77] Perner F. Stein E. M. Wenge D. V. Singh S. Kim J. Apazidis A. (2023). MEN1 mutations mediate clinical resistance to menin inhibition. Nature 615 (7954), 913–919. 10.1038/s41586-023-05755-9 36922589 PMC10157896

[B78] Pirozzi C. J. Yan H. (2021). The implications of IDH mutations for cancer development and therapy. Nat. Rev. Clin. Oncol. 18 (10), 645–661. 10.1038/s41571-021-00521-0 34131315

[B79] Poddar S. K. Yang Y. Pal P. Jin Z. Pei J. Xiao Y. (2025). Discovery of dual BCL-xL/BCL-w degraders by exploiting the bis (sulfonyl) benzene ring of ABT-263 as a linkage vector. J. Med. Chem. 68, 18684–18702. 10.1021/acs.jmedchem.5c01834 40880408 PMC12550731

[B80] Quintás-Cardama A. Hu C. Qutub A. Qiu Y. Zhang X. Post S. (2017). p53 pathway dysfunction is highly prevalent in acute myeloid leukemia independent of TP53 mutational status. Leukemia 31 (6), 1296–1305. 10.1038/leu.2016.350 27885271

[B81] Rego E. M. De Santis G. C. (2011). Differentiation syndrome in promyelocytic leukemia: clinical presentation, pathogenesis and treatment. Mediterr. J. Hematol. Infect. Dis. 3 (1), e2011048. 10.4084/MJHID.2011.048 22110898 PMC3219650

[B82] Roberts A. W. Wei A. H. Huang D. C. (2021). BCL2 and MCL1 inhibitors for hematologic malignancies. Blood, J. Am. Soc. Hematol. 138 (13), 1120–1136. 10.1182/blood.2020006785 34320168

[B83] Salamero O. Molero A. Pérez-Simón J. A. Arnan M. Coll R. Garcia-Avila S. (2024). Iadademstat in combination with azacitidine in patients with newly diagnosed acute myeloid leukaemia (ALICE): an open-label, phase 2a dose-finding study. Lancet Haematol. 11 (7), e487–e498. 10.1016/S2352-3026(24)00132-7 38824932

[B84] Schüpbach A. Akhoundova D. Bacher U. Nilius H. Hoffmann M. Largiadèr C. R. (2025). Impact of venetoclax treatment schedule on hematologic recovery and treatment response in AML patients unfit for intensive chemotherapy. Cancers (Basel) 17 (7), 1138. 10.3390/cancers17071138 40227639 PMC11987944

[B85] Seiler M. Yoshimi A. Darman R. Chan B. Keaney G. Thomas M. (2018). H3B-8800, an orally available small-molecule splicing modulator, induces lethality in spliceosome-mutant cancers. Nat. Med. 24 (4), 497–504. 10.1038/nm.4493 29457796 PMC6730556

[B86] Shah N. P. Bhatia R. Altman J. K. Amaya M. Begna K. H. Berman E. (2024). NCCN clinical practice guidelines in oncology: chronic myeloid leukemia (version 2.2024). J. Natl. Compr. Cancer Netw. (JNCCN) 22 (1), 43–69. 10.6004/jnccn.2024.0007 38394770

[B87] Sharzehi S. Joshi S. K. Pittsenbarger J. Tyner J. W. Traer E. (2021). The FLT3 F691L gatekeeper mutation promotes clinical resistance to gilteritinib+ venetoclax (GILT+ VEN) in AML. Blood 138, 2235. 10.1182/blood-2021-145762

[B88] Shastri A. Goldfinger M. Mantzaris I. Garcia-Manero G. Kadia T. M. Ning J. (2024). A phase 1 study investigating the safety and efficacy of danvatirsen as monotherapy followed by combination with venetoclax in patients with relapsed/refractory MDS and AML. Blood 144, 4265.5. 10.1182/blood-2024-208998

[B89] Short N. J. Daver N. Dinardo C. D. Kadia T. Nasr L. F. Macaron W. (2024). Azacitidine, venetoclax, and gilteritinib in newly diagnosed and relapsed or refractory FLT3-mutated AML. J. Clin. Oncol. 42 (13), 1499–1508. 10.1200/JCO.23.01911 38277619 PMC11095865

[B90] Singh A. Bosma G. Zhang J. Abbott D. Amaya M. Kent A. (2025). Outcomes for adults with KMT2A-r acute myeloid leukemia treated with venetoclax plus azacitidine (Ven/Aza) or intensive chemotherapy (IC): a single-center retrospective study. American Society of Clinical Oncology. 43 (suppl. 16), e18537. 10.1200/JCO.2025.43.16_suppl.e18537

[B91] Smith C. C. Levis M. J. Perl A. E. Hill J. E. Rosales M. Bahceci E. (2022). Molecular profile of FLT3-mutated relapsed/refractory patients with AML in the phase 3 ADMIRAL study of gilteritinib. Blood Adv. 6 (7), 2144–2155. 10.1182/bloodadvances.2021006489 35130342 PMC9006281

[B92] Song Y. Bi Z. Liu Y. Qin F. Wei Y. Wei X. (2023). Targeting RAS–RAF–MEK–ERK signaling pathway in human cancer: current status in clinical trials. Genes Dis. 10 (1), 76–88. 10.1016/j.gendis.2022.05.006 37013062 PMC10066287

[B93] Staehle H. F. Koellerer C. Staehle A. M. Schulze J. Eble P. Müller A. (2025). Lysine-specific demethylase 1 regulates hematopoietic stem cell expansion and myeloid cell differentiation. Cell Death Dis. 16 (1), 619. 10.1038/s41419-025-07951-z 40813574 PMC12354751

[B94] Stanley R. F. Abdel-Wahab O. (2022). Dysregulation and therapeutic targeting of RNA splicing in cancer. Nat. Cancer 3, 536–546. 10.1038/s43018-022-00384-z 35624337 PMC9551392

[B95] Steens J. Klein D. (2022). HOX genes in stem cells: maintaining cellular identity and regulation of differentiation. Front. Cell Dev. Biol. 10, 1002909. 10.3389/fcell.2022.1002909 36176275 PMC9514042

[B96] Steensma D. P. Wermke M. Klimek V. M. Greenberg P. L. Font P. Komrokji R. S. (2021). Phase I first-in-human dose escalation study of the oral SF3B1 modulator H3B-8800 in myeloid neoplasms. Leukemia 35 (12), 3542–3550. 10.1038/s41375-021-01328-9 34172893 PMC8632688

[B97] Stein E. M. DiNardo C. D. Pollyea D. A. Fathi A. T. Roboz G. J. Altman J. K. (2017). Enasidenib in mutant IDH2 relapsed or refractory acute myeloid leukemia. Blood, J. Am. Soc. Hematol. 130 (6), 722–731. 10.1182/blood-2017-04-779405 28588020 PMC5572791

[B98] Steinberg-Shemer O. Orenstein N. Krasnov T. Noy-Lotan S. Marcoux N. Dgany O. (2022). Congenital thrombocytopenia associated with a heterozygous variant in the MEIS1 gene encoding a transcription factor essential for megakaryopoiesis. Platelets 33 (4), 645–648. 10.1080/09537104.2021.1961704 35130804

[B99] Stone R. M. Mandrekar S. J. Sanford B. L. Laumann K. Geyer S. Bloomfield C. D. (2017). Midostaurin plus chemotherapy for acute myeloid leukemia with a FLT3 mutation. N. Engl. J. Med. 377 (5), 454–464. 10.1056/NEJMoa1614359 28644114 PMC5754190

[B100] Sweet K. Cluzeau T. (2025). Clinical perspectives on post‐induction maintenance therapy in patients with acute myeloid leukaemia in remission who are ineligible for allogeneic haematopoietic stem cell transplantation. Br. J. Haematol. 206 (1), 61–68. 10.1111/bjh.19924 39622271 PMC11739750

[B101] Takao S. Morell V. Uni M. Slavit A. Rha S. Cheng S. (2025). Epigenetic mechanisms controlling human leukemia stem cells and therapy resistance. Nat. Commun. 16 (1), 3196. 10.1038/s41467-025-58370-9 40180954 PMC11968996

[B102] Thier J. Hofmann S. Kirchhof K. M. Todisco G. Mortera-Blanco T. Lilienthal I. (2025). SF3B1-mutant models of RNA mis-splicing uncover UBA1 as a therapeutic target in myelodysplastic neoplasms: myelodysplastic neoplasm. Leukemia, 1–11. 10.1038/s41375-025-02740-1 40858805 PMC12589102

[B103] Venhuizen J. van Bergen M. G. J. M. Bergevoet S. M. Gilissen D. Spruijt C. G. Wingens L. (2024). GFI1B and LSD1 repress myeloid traits during megakaryocyte differentiation. Commun. Biol. 7 (1), 374. 10.1038/s42003-024-06090-z 38548886 PMC10978956

[B104] Vervloessem T. Ivanova H. Luyten T. Parys J. B. Bultynck G. (2017). The selective Bcl-2 inhibitor venetoclax, a BH3 mimetic, does not dysregulate intracellular Ca^2+^ signaling. Biochimica Biophysica Acta (BBA)-Molecular Cell Res. 1864 (6), 968–976. 10.1016/j.bbamcr.2016.11.024 27913204

[B105] Vom Stein A. F. Frenzel L. P. (2025). Understanding and targeting BCL2-inhibitor resistance in chronic lymphocytic leukemia. Hematology/Oncology Clin. 39 (5), 965–979. 10.1016/j.hoc.2025.06.001 40695714

[B106] Wang F. Morita K. DiNardo C. D. Furudate K. Tanaka T. Yan Y. (2021). Leukemia stemness and co-occurring mutations drive resistance to IDH inhibitors in acute myeloid leukemia. Nat. Commun. 12 (1), 2607. 10.1038/s41467-021-22874-x 33972549 PMC8110775

[B107] Wang P. Zhang Y. Xiang R. Yang J. Xu Y. Deng T. (2024a). Foretinib is effective in acute myeloid leukemia by inhibiting FLT3 and overcoming secondary mutations that drive resistance to quizartinib and gilteritinib. Cancer Res. 84 (6), 905–918. 10.1158/0008-5472.CAN-23-1534 38231480 PMC10940854

[B108] Wang E. S. Issa G. C. Erba H. P. Altman J. K. Montesinos P. DeBotton S. (2024b). Ziftomenib in relapsed or refractory acute myeloid leukaemia (KOMET-001): a multicentre, open-label, multi-cohort, phase 1 trial. Lancet Oncol. 25 (10), 1310–1324. 10.1016/S1470-2045(24)00386-3 39362248

[B109] Wang Z. Lai R. Wang X. Chen X. Zhou Y. Li S. (2025a). Targeted penetrating motif engineering of BH3 mimetic: harnessing non‐canonical amino acids for coinhibition of MCL‐1 and BCL‐xL in acute myeloid leukemia. Adv. Sci. 12, 2503682. 10.1002/advs.202503682 40305693 PMC12279208

[B110] Wang X. Yang W. Shen B. Jiang B. Zhang Z. Wu H. (2025b). Nanomedicine targeting the Warburg effect: advanced strategies for cancer therapy. Crit. Rev. Oncology/Hematology 215, 104922. 10.1016/j.critrevonc.2025.104922 40912591

[B111] Wei D. Wang L. Zuo X. Maitra A. Bresalier R. S. (2024). A small molecule with big impact: MRTX1133 targets the KRASG12D mutation in pancreatic cancer. Clin. Cancer Res. 30 (4), 655–662. 10.1158/1078-0432.CCR-23-2098 37831007 PMC10922474

[B112] Willekens C. Bazinet A. Chraibi S. Bataller A. Decroocq J. Arani N. (2025). Reduced venetoclax exposure to 7 days vs standard exposure with hypomethylating agents in newly diagnosed AML patients. Blood Cancer J. 15 (1), 68. 10.1038/s41408-025-01269-x 40246832 PMC12006504

[B113] Wu S.-J. Kuo Y.-Y. Hou H.-A. Li L.-Y. Tseng M.-H. Huang C.-F. (2012). The clinical implication of SRSF2 mutation in patients with myelodysplastic syndrome and its stability during disease evolution. Blood, J. Am. Soc. Hematol. 120 (15), 3106–3111. 10.1182/blood-2012-02-412296 22932795

[B114] Wysota M. Konopleva M. Mitchell S. (2024). Novel therapeutic targets in acute myeloid leukemia (AML). Curr. Oncol. Rep. 26 (4), 409–420. 10.1007/s11912-024-01503-y 38502417 PMC11021231

[B115] Xiao K. Zhang Z. Wu Y. Li G. Chen J. Ren Y. (2025). Discovery of HMPL-306 (Ranosidenib), a new potent and selective dual inhibitor of mutant IDH1 and 2 in clinical development for cancer treatment. ACS Med. Chem. Lett. 16 (3), 454–463. 10.1021/acsmedchemlett.4c00625 40104804 PMC11912272

[B116] Yamatani K. Carter B. Z. Konopleva M. Tabe Y. Andreeff M. (2021). AML-173: BCL2A1: a novel target in refractory/relapsed AML with FLT3-ITD/D835 double mutations. Clin. Lymphoma Myeloma Leukemia 21, S286–S287. 10.1016/s2152-2650(21)01687-6

[B117] Yoshimi A. Lin K.-T. Wiseman D. H. Rahman M. A. Pastore A. Wang B. (2019). Coordinated alterations in RNA splicing and epigenetic regulation drive leukaemogenesis. Nature 574 (7777), 273–277. 10.1038/s41586-019-1618-0 31578525 PMC6858560

[B118] Zeidner J. F. Lin T. L. Welkie R. L. Curran E. Koenig K. Stock W. (2025a). Azacitidine, venetoclax, and revumenib for newly diagnosed NPM1-mutated or KMT2A-rearranged AML. J. Clin. Oncol. 43, 2606–2615. 10.1200/JCO-25-00914 40504618 PMC12316144

[B119] ZeidnerJ. F. SallmanD. A. RécherC. DaverN. G. LeungA. Y. H. HiwaseD. K. (2025b). Magrolimab plus azacitidine vs physician’s choice for untreated TP53-mutated acute myeloid leukemia: the ENHANCE-2 study. Blood 146 (5), 590–600. 10.1182/blood.2024027408 40009500

[B120] Zeng C. Nie D. Wang X. Zhong S. Zeng X. Liu X. (2024). Combined targeting of GPX4 and BCR-ABL tyrosine kinase selectively compromises BCR-ABL+ leukemia stem cells. Mol. Cancer 23 (1), 240. 10.1186/s12943-024-02162-0 39465372 PMC11514791

[B121] Zhang Y. Qian J. Gu C. Yang Y. (2021). Alternative splicing and cancer: a systematic review. Sig Transduct. Target Ther. 6, 78. 10.1038/s41392-021-00486-7 33623018 PMC7902610

[B122] Zhang K. Zhang X. Xu Y. Xue S. Qiu H. Tang X. (2023). Efficacy of venetoclax combined with hypomethylating agents in young, and unfit patients with newly diagnosed core binding factor acute myeloid leukemia. Blood Cancer J. 13 (1), 155. 10.1038/s41408-023-00928-1 37821435 PMC10567686

[B123] Zhang J. Xu Y. Lin Z. Chen J. Liu L. Wu D. (2024). Efficacy and safety of venetoclax in combination with hypomethylating agents for the treatment of high-risk myelodysplastic syndromes–real-world analyses. Blood 144 (Suppl. 1), 6732. 10.1182/blood-2024-199990 40017202

