# Supplementary material for: Acute myeloid leukemia drug resistance: targetable nodes and the clinical trajectory of small-molecule inhibitors
Source: Front Pharmacol. 2025 Nov 19;16:1696229. doi: 10.3389/fphar.2025.1696229 (PMC12672468; doi:10.3389/fphar.2025.1696229)
Supplement: Supplementary file 1 [file Supplementaryfile1.docx]

Glossary

LSC (Leukemic Stem Cell): A subpopulation of leukemic cells with self-renewal and disease-initiating capabilities, often driving therapy resistance and relapse in AML.

NGS (Next-Generation Sequencing): High-throughput DNA sequencing technology that enables rapid detection of mutations, insertions, deletions, and fusions in AML, supporting genetic profiling and targeted therapy selection.

FLT3 (FMS-Like Tyrosine Kinase 3): A receptor tyrosine kinase gene; mutations (e.g., internal tandem duplication [ITD], point mutations like D835Y) are common in AML, with FLT3-ITD linked to poor prognosis.

IDH1/2 (Isocitrate Dehydrogenase 1/2): Genes encoding metabolic enzymes; mutations in AML result in production of the oncometabolite 2-hydroxyglutarate, disrupting cell differentiation and amenable to targeted inhibition.

MLL/KMT2A (Mixed Lineage Leukemia / Lysine Methyltransferase 2A): A gene involved in histone methylation; rearrangements (e.g., MLL-AF9) in AML are linked to aggressive disease and poor outcomes, especially in infants.

HMAs (Hypomethylating Agents): A class of drugs (e.g., azacitidine, decitabine) that inhibit DNA methyltransferases, reversing aberrant DNA methylation in AML; commonly used in elderly or unfit patients.

DS (Differentiation Syndrome): A life-threatening complication of differentiation-inducing therapies (e.g., venetoclax, ATRA) in AML, caused by rapid blast maturation and cytokine release, leading to organ dysfunction.

ctDNA (Circulating Tumor DNA): Fragmented DNA released by tumor cells into the bloodstream; in AML, ctDNA can be used for non-invasive detection of minimal residual disease (MRD), monitoring clonal evolution, and predicting relapse.

MRD (Minimal Residual Disease): Low levels of residual leukemic cells in AML patients who achieved complete remission (CR); undetectable by conventional morphology, but detectable via sensitive techniques (e.g., NGS, flow cytometry), and a strong predictor of relapse.

BH3 Mimetics: A class of targeted drugs that mimic BH3-only proteins, binding to anti-apoptotic BCL-2 family members (e.g., BCL-2) to induce leukemic cell apoptosis; e.g., venetoclax.

Venetoclax: An oral selective BCL-2 inhibitor (a BH3 mimetic) used in AML, particularly in elderly/unfit patients or those with relapsed/refractory disease, by reactivating the apoptotic pathway.

DNA Methylation: An epigenetic modification involving methyl group addition to DNA, which represses gene transcription; dysregulated DNA methylation is a key hallmark of AML, targeted by HMAs.

ATR (Ataxia Telangiectasia and Rad3 Related Kinase): A key component of the DNA damage response (DDR) pathway that regulates cell cycle checkpoints; in BH3-targeted therapy, ATR inhibitors are used to downregulate MCL-1 and amplify replication stress, with administration timed to avoid overlapping myelosuppressive nadirs.​

BCL-XL (B-Cell Lymphoma Extra-Large): An anti-apoptotic protein in the BCL-2 family; BH3-targeted therapies targeting BCL-XL (e.g., BCL-XL degraders) are often used in venetoclax-experienced patients, with platelet safety gates (≥50×10^9/L) to minimize hemorrhagic risk.​

BH3 Profiling: A functional assay used to assess the dependency of cancer cells on anti-apoptotic proteins (e.g., BCL-2, MCL-1, BCL-XL) by measuring mitochondrial sensitivity to BH3-only peptides; in this review, it serves as a predictive biomarker to guide patient enrichment in early-phase trials.​

BH3 Shift: A pharmacodynamic readout indicating successful target engagement of BH3-targeted therapies (e.g., reduced dependency on MCL-1/BCL-XL); lack of BH3 shift by cycle 2 is a pre-specified stopping rule in clinical trials.​

DDR (DNA Damage Response): A cellular pathway that detects and repairs DNA damage; integration of DDR modules (e.g., ATR, WEE1 inhibitors) with BH3 pulses enhances therapeutic efficacy by downregulating MCL-1 and amplifying replication stress.​

DoR (Duration of Response): A clinical endpoint measuring the length of time a patient maintains a response to treatment; in BH3-targeted therapy trials, it is evaluated alongside overall response rate (ORR) and early MRD conversion.

MCL-1 (Myeloid Cell Leukemia 1): An anti-apoptotic protein in the BCL-2 family, a key mediator of resistance to venetoclax; short-pulse MCL-1 inhibitors are paired with venetoclax in venetoclax-experienced patients, with a cardio-protection bundle (e.g., 48–72 h telemetry) to manage cardiac risk.

MEK/ERK (Mitogen-Activated Protein Kinase Kinase/Extracellular Signal-Regulated Kinase): A signaling pathway activated in inflammatory or RAS-mutant AML; MEK/ERK inhibitors (e.g., trametinib) are added to BH3-based combinations to suppress stress-induced MCL-1/BCL-XL upregulation.

ORR (Overall Response Rate): A clinical endpoint measuring the proportion of patients achieving a complete or partial response to treatment; in BH3-targeted therapy trials, it is complemented by endpoints like early MRD conversion and failure-free survival.​

PD (Pharmacodynamic): Relating to the effects of a drug on the body; in-cycle PD readouts (e.g., time-to-MOMP, MCL-1 protein decline) are used to confirm target engagement and guide dose adjustment in BH3-targeted therapy.

Thrombocytopenia: An abnormally low platelet count (<150×10⁹/L) in AML, caused by impaired platelet production; increases the risk of bleeding (e.g., petechiae, hemorrhage).

Neutropenia: An abnormally low neutrophil count (<2.0×10⁹/L); severe neutropenia (<0.5×10⁹/L) in AML (often post-chemotherapy) increases susceptibility to life-threatening bacterial/fungal infections.

IVO (IDH1 Inhibitor, e.g., Ivosidenib): A class of small molecule drugs that specifically target mutated Isocitrate Dehydrogenase 1 (IDH1). In AML, IDH1 mutations lead to the production of the oncometabolite 2-hydroxyglutarate. Inhibitors like ivosidenib block this mutant enzyme, reversing the abnormal cell differentiation process. Approved by the FDA for both newly diagnosed and relapsed/refractory IDH1-mutated AML patients. In clinical trials such as the AGILE study, IVO combined with azacitidine significantly improved overall survival (29.3 months vs 7.9 months) and response rates in unfit newly diagnosed IDH1-mutated AML patients compared to placebo plus azacitidine.

ENA (IDH2 Inhibitor, e.g., Enasidenib): Small molecule inhibitors that target mutated Isocitrate Dehydrogenase 2 (IDH2). IDH2 mutations also result in the production of 2-hydroxyglutarate, disrupting normal hematopoiesis. Enasidenib has shown efficacy in treating relapsed/refractory IDH2-mutated AML, leading to remissions in a subset of patients. It works by inhibiting the mutant IDH2 enzyme, restoring normal cell function and differentiation.

FLT3 Inhibitor (e.g., Gilteritinib, Midostaurin): These small molecules target the FMS-like Tyrosine Kinase 3 (FLT3) receptor, which is frequently mutated in AML. FLT3 mutations, especially internal tandem duplication (ITD) mutations, are associated with a poor prognosis. Inhibitors such as gilteritinib and midostaurin block the hyperactive signaling pathway of mutant FLT3, inhibiting leukemic cell growth and survival. Midostaurin was the first FLT3 inhibitor approved for newly diagnosed FLT3-mutated AML in combination with chemotherapy, improving survival outcomes. Gilteritinib is approved for relapsed/refractory FLT3-mutated AML and has shown activity in single-agent treatment, targeting both FLT3-ITD and tyrosine kinase domain (TKD) mutations.

Menin Inhibitor (e.g., SNDX-5613 (Revumenib), KO-539 (Ziftomenib), BN104): Menin inhibitors target the interaction between menin and lysine methyltransferase 2A (KMT2A), which is crucial for the pathogenesis of AML with KMT2A rearrangement or Nucleophosmin 1 (NPM1) mutation. In AML patients with KMT2A rearrangement (occurring in 5-10% of cases) or NPM1 mutation (20-30% of cases), these inhibitors disrupt the abnormal gene expression programs. Clinical trials with menin inhibitors like Revumenib and Ziftomenib have shown response rates of approximately 30% in NPM1-mutated patients, 27% with Revumenib and 5.6% with Ziftomenib in KMT2A-rearranged leukemias. BN104, in 2024 ASH data, showed a complete remission/complete remission with partial hematologic recovery rate of 60.9% in the KMT2A-rearranged subgroup and 40% in the NPM1-mutated subgroup of relapsed/refractory AML patients, with tolerable safety.


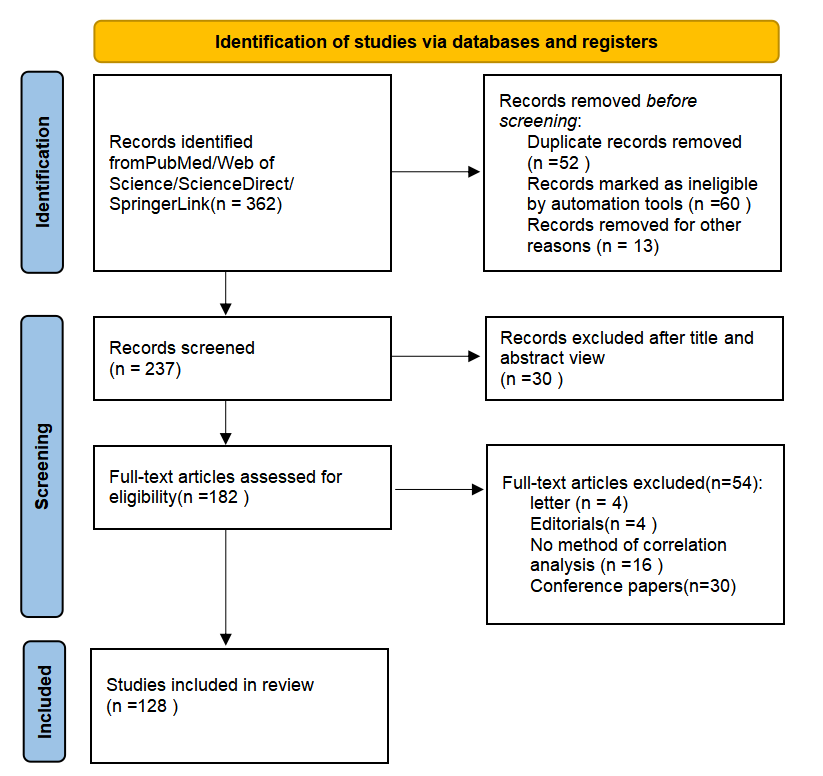


Figure 1. PRISMA flow diagram of study selection for the systematic review on acute myeloid leukemia drug resistance.
